# Experimental and bioinformatic characterization of a recombinant polygalacturonase-inhibitor protein from pearl millet and its interaction with fungal polygalacturonases

**DOI:** 10.1093/jxb/eru266

**Published:** 2014-06-30

**Authors:** S. Ashok Prabhu, Ratna Singh, Stephan Kolkenbrock, Neerakkal Sujeeth, Nour Eddine El Gueddari, Bruno M. Moerschbacher, Ramachandra K. Kini, Martin Wagenknecht

**Affiliations:** ^1^Department of Studies in Biotechnology, University of Mysore, Manasagangotri, Mysore-570 006, Karnataka, India; ^2^Institut für Biologie und Biotechnologie der Pﬂanzen, Westfälische Wilhelms-Universität Münster, Schlossplatz 8, D-48143 Münster, Germany; ^3^Molecular Biology of Plants, Groningen Biomolecular Sciences and Biotechnology Institute, Centre for Life Sciences, University of Groningen, Nijenborgh 7, 9747 AG Groningen, The Netherlands

**Keywords:** Computational mutagenesis, electrostatic surface potential, inhibition studies, pearl millet, *Phaseolus vulgaris*, PGIPs, PGs, protein modelling and docking.

## Abstract

Polygalacturonases (PGs) are hydrolytic enzymes employed by several phytopathogens to weaken the plant cell wall by degrading homopolygalacturonan, a major constituent of pectin. Plants fight back by employing polygalacturonase-inhibitor proteins (PGIPs). The present study compared the inhibition potential of pearl millet PGIP (*Pennisetum glaucum*; *Pgl*PGIP1) with the known inhibition of *Phaseolus vulgaris* PGIP (*Pv*PGIP2) against two PGs, the PG-II isoform from *Aspergillus niger* (*An*PGII) and the PG-III isoform from *Fusarium moniliforme* (*Fm*PGIII). The key rationale was to elucidate the relationship between the extent of sequence similarity of the PGIPs and the corresponding PG inhibition potential. First, a pearl millet *pgip* gene (*Pglpgip1*) was isolated and phylogenetically placed among monocot PGIPs alongside foxtail millet (*Setaria italica*). Upstream sequence analysis of *Pglpgip1* identified important *cis*-elements responsive to light, plant stress hormones, and anoxic stress. *Pgl*PGIP1, heterologously produced in *Escherichia coli*, partially inhibited *An*PGII non-competitively with a pH optimum between 4.0 and 4.5, and showed no inhibition against *Fm*PGIII. Docking analysis showed that the concave surface of *Pgl*PGIP1 interacted strongly with the N-terminal region of *An*PGII away from the active site, whereas it weakly interacted with the C-terminus of *Fm*PGIII. Interestingly, *Pgl*PGIP1 and *Pv*PGIP2 employed similar motif regions with few identical amino acids for interaction with *An*PGII at non-substrate-binding sites; however, they engaged different regions of *An*PGII. Computational mutagenesis predicted D126 (*Pgl*PGIP1)–K39 (*An*PGII) to be the most significant binding contact in the *Pgl*PGIP1–*An*PGII complex. Such protein–protein interaction studies are crucial in the future generation of designer host proteins for improved resistance against ever-evolving pathogen virulence factors.

## Summary

We undertook production and inhibition studies of recombinant millet PGIP. Using computational mutagenesis, the most significant binding contact involved in pearl millet PGIP–AnPGII interaction was identified.

## Introduction

Pectin, a galacturonic acid-rich complex polysaccharide found in all plant cell walls (CWs), is composed of homogalacturonan (HG), xylogalacturonan, apiogalacturonan, rhamnogalacturonan I, and rhamnogalacturonan II (Vorwerk *et al.*, 2004). HG, a linear homopolymer of α-1,4-linked d-galactopyranosyluronic acid with varying extents of methylation and acetylation, is the most abundant component (Mohnen, 2008). Phytopathogens are known to produce endo*-* and exo-polygalacturonases (PGs) that can breakdown HG (Van den Brink and De Vries, 2011). As a counter stratagem, plants employ polygalacturonase-inhibitor proteins (PGIPs) to modulate PG activity leading to an accumulation of elicitor-active oligogalacturonides (De Lorenzo *et al.*, 2001). PGIPs are CW-bound glycoproteins belonging to the extracyoplasmic leucine-rich repeat (LRR) family of proteins (Shanmugam, 2005). PGIPs are very diverse in their PG-inhibition specificity and potential with varying degrees of inhibition (Gomathi and Gnanamanickam, 2004).

PG–PGIP complexes are considered a model protein–protein interaction system in the backdrop of plant–pathogen interactions (Misas-Villamil and Van der Hoorn, 2008). Although three-dimensional structures of many PGs have been elucidated to date (Pickersgill *et al.*, 1998; Federici *et al.*, 1999; Bonivento *et al.*, 2008), the only PGIP whose crystal structure has been solved is that of *Pv*PGIP2 from *Phaseolus vulgaris* (Di Matteo *et al.*, 2003). Most of the data available on the PG–PGIP interactions has been a result of studies involving *Pv*PGIP2. Previous studies employed targeted mutation of *pg* and *pgip* genes, and investigated the *in vitro* inhibition behaviour of the protein variants synthesized to identify the amino acid residues involved in the protein–protein interactions (Leckie *et al.*, 1999; Mattei *et al.*, 2001; Raiola *et al.*, 2008). Amide-exchange mass spectrometry in combination with protease protection and fluorescence spectrometric analysis was employed to deduce the amino acids of *An*PGII, a PG isoform II from *Aspergillus niger*, required for interaction with *Pv*PGIP2 (King *et al.*, 2002). The availability of advanced bioinformatic tools for protein homology modelling and docking have been exploited in in-depth analysis of PG–PGIP complexes *in silico* and found to be in conformity with the experimental results (Lim *et al.*, 2009; Maulik *et al.*, 2009).

In contrast to the magnitude of literature available on dicot PGIPs, information available in case of monocots is meagre. Although PGIPs from wheat and rice have been tested for inhibition against various PGs (Jang *et al.*, 2003; Kemp *et al.*, 2003; Janni *et al.*, 2006, 2013), no efforts have gone into understanding their mode of inhibition and the underlying structural basis of their interaction with PGs. In addition, no attempt has been made towards characterization of PGIPs from millets, small-grained gramineous monocots. Pearl millet [*Pennisetum glaucum* (L.) R. Br.; synonym: *Cenchrus americanus* (L.) Morrone], is among the most important cereal crops grown in the semi-arid tropical regions of Africa and the Indian subcontinent (Sehgal *et al.*, 2012).

In the present study, the gene encoding pearl millet PGIP was isolated and expressed heterologously as a maltose-binding protein (MBP) fusion in *Escherichia coli*. The purified recombinant fusion protein was employed in *in vitro* inhibition studies against two fungal PGs, *An*PGII and *Fm*PGIII (PG isoform III from *Fusarium moniliforme* isolate PD). *Pv*PGIP2, the most wide-spectrum and potent inhibitor of fungal PGs (Farina *et al.*, 2009), has been shown to inhibit *An*PGII (Stotz *et al.*, 2000) and is reported to be ineffective against *Fm*PGIII (Sella *et al.*, 2004). A study of the inhibition profile of pearl millet PGIP against the same two PGs was carried out to elucidate the relationship between the extent of sequence similarity and the corresponding ability to inhibit PG. Furthermore, in the present study, *in silico* protein modelling, docking, and mutation analyses were carried out to explain the *in vitro* results, gain an understanding of the underlying structural basis of interaction, and predict the putative amino acids involved. To the best of our knowledge, this is the first report on the production of recombinant PGIP from millets and exploration of its inhibitory potential.

## Materials and methods

### Plant material

Seeds of pearl millet (*P. glaucum*) cultivar IP18296, obtained from the International Crops Research Institute for the Semi-Arid Tropics, Hyderabad, India, were used in this study. The seeds were germinated on moist germination sheets; 2-d-old seedlings were harvested and stored at –80 °C till further use.

### Isolation of nucleic acids

Total RNA and genomic DNA were extracted from 2-d-old pearl millet coleoptiles using a Total Plant RNA Isolation kit (Sigma) and a GeneJET™ Plant Genomic DNA Purification Mini kit (Thermo Scientific), respectively, as per the manufacturer’s instructions.

### *Isolation and cloning of partial* pgip *genes*

The cDNA was prepared by means of a two-step AMV RT-PCR kit (Qiagen) as per the manufacturer’s instructions, using total RNA from pearl millet coleoptiles as template. For PCR amplification of the partial *pgip* genes, primers Par1For (5ʹ-CTCGACCTCTCCTTCAACTC-3ʹ)/Par1Rev (5ʹ-ATGCCGCC GTAGATGGCGTT GTG-3ʹ) and Par2For (5ʹ-TGCGACTGGTA CGACGTCGACTG-3ʹ)/ Par2Rev (5ʹ-TCGCCACCTGCGCCGGG ATG-3ʹ) were designed based on the consensus region obtained by alignment of known monocot *pgip* gene sequences (GenBank accession nos AM180652–AM180657, NP_001147231, and XP_002439099) (Clone Manager Professional 9, Sci-Ed software). The amplification using *Taq* DNA polymerase (Merck Biosciences) resulted in ~400bp (*Pglpgip1p*) and ~500bp (*Pglpgip2p*) bands, respectively. They were gel purified using a QIAquick Gel Extraction kit (Qiagen), cloned in pTZ57R/T (InsTA clone PCR Cloning kit; Fermentas), and sequenced at Eurofins MWG Operon, Germany. Nucleotide BLAST (http://blast.ncbi.nlm.nih.gov/Blast.cgi) searches were performed using default parameters to ascertain the gene identity.

### Southern blot analysis

Pearl millet genomic DNA (10 µg) was digested with restriction endonucleases separately: *Apo*I (New England Biolabs, Germany), *Ahd*I, *Bam*HI, *Eco*RI, *Hin*dIII, *Kpn*I, *Msc*I, *Nae*I, *Sac*I, *Xba*I, and *Xho*I (Thermo Scientific) and separated electrophoretically on a 0.7% agarose gel as described by Sambrook and Russell (2001). The DNA was transferred to a positively charged nylon membrane (Roche) as per the manufacturer’s instructions. As a hybridization probe, PCR-amplified *Pglpgip1p* was random-prime labelled using a DIG High Prime DNA Labeling and Detection Starter kit II (Roche). Pre-hybridization, hybridization, and chemiluminescent detection were performed as described previously (Wagenknecht and Meinhardt, 2011). Hybridization was carried out at 63 °C.

### Inverse PCR

Pearl millet genomic DNA (3 µg) was digested using *Apo*I (New England Biolabs) and the digest mix was cleaned up using a NucleoSpin^®^ Gel and PCR Clean-up kit (Macherey-Nagel). A 500ng aliquot of the cleaned-up digest was self-ligated using a Rapid DNA Ligation kit (Thermo Scientific).

The following two sets of inverse PCR primers, Inv1A (5ʹ-AC GCCTTCAGCTTCAACCTCTC-3ʹ)/Inv1B (5ʹ-TTGTGCGACA GCACTAGGGATG-3ʹ) and Inv2A (5ʹ-AGAGGTAGATCTGGT CGGCG-3ʹ)/Inv2B (5ʹ-CGCCAATTTCGCGCACC-3ʹ) were designed based on the *Pglpgip1p* and *Pglpgip2p* sequences, respectively. The PCR was carried out using Phusion^®^ Hot Start High-Fidelity DNA polymerase (Finnzymes) with 30ng of self-ligated DNA as template. To the blunt-end PCR product of ~2kb obtained with Inv1A/Inv1B, an ‘A’ overhang was attached using *Taq* DNA polymerase (Thermo Scientific). It was further cloned in the pGEM-T Easy vector (Promega) and sequenced at Eurofins MWG Operon, Germany.

### Sequence assembly

Sequence assembly (Clone Manager Professional 9, Sci-Ed software) was carried out using sequences obtained by inverse PCR and the *Pglpgip1p* sequence to determine the open reading frame (ORF) (*Pglpgip1*). Nucleotide BLAST searches were performed to ascertain the gene identity.

### *Bioinformatic analysis of* Pglpgip1

The deduced amino acid sequence of *Pglpgip1* (*Pgl*PGIP1) was further subjected to various *in silico* analyses using different bioinformatics tools: the signal peptide was identified using SignalP 4.1 (Petersen *et al.*, 2011) and the targeted protein localization was predicted using WoLF PSORT (Nakai and Horton, 1999). The assignment of domains in *Pgl*PGIP1 was performed based on the NCBI conserved domain search analysis (Marchler-Bauer *et al.*, 2011) and on a comparative amino acid sequence alignment with *Pv*PGIP2 domain architecture (Di Matteo *et al.*, 2003) using the T-Coffee tool (Notredame *et al.*, 2000). The putative N-glycosylation sites were determined using NetNGly 1.0 (http://www.cbs.dtu.dk/services/NetNGlyc/).

The *Pgl*PGIP1 and other known monocot and dicot PGIP sequences (protein accession numbers are summarized in Supplementary Table S1 at *JXB* online) from the National Center for Biotechnology Information database were aligned with MUSCLE version 3.7 (Edgar *et al.*, 2004) and gaps and/or poorly aligned regions were removed with Gblocks version 0.91b (Talavera and Castresana, 2007). A phylogenetic tree was generated using the maximum-likelihood method employed in PhyML version 3.0 aLRT (Anisimova and Gascuel, 2006) on the http://www.phylogeny.fr platform (Dereeper *et al.*, 2008) using default settings. Internal branch consistency was appraised using the bootstrapping method with 100 bootstrap replicates. The tree was rendered using TreeDyn version 198.3 (Chevenet *et al.*, 2006).

The nucleotide sequence upstream of the ORF was submitted to PlantCARE (Lescot *et al.*, 2002) to determine the presence of plant-specific *cis*-elements.

### *Construction of the* Pgl*PGIP1 and* Fm*PGIII expression plasmids*

The multistep cloning strategy involved in construction of the *Pgl*PGIP1 expression plasmid (pET-22b::MBP-IEGR-*Pgl*PGIP1-6×His-Strep-tag^®^ II, where IEGR is the factor Xa protease cleavage site and 6×His is a hexa-histidine tag) (construct E), vector control (pET-22b::MBP-IEGR-6×His-Strep-tag^®^ II) (construct F) and the *Fm*PGIII expression (pET-22b::*Fm*PGIII-Strep-tag^®^ II) (construct G) plasmids have been detailed in Supplementary Table S2 at *JXB* online (Supplementary Fig. S1 at *JXB* online provides a pictorial representation of the constructs). Briefly, the *Pglpgpip1* coding sequence was initially cloned in pET-22b(+) in frame with the vector-encoded 6×His sequence. Then, *malE* (MBP-encoding gene), followed by a factor Xa protease recognition site was cloned upstream of *pgip*, and a Strep-tag^®^ II-encoding sequence was cloned downstream of 6×His, allowing the synthesis of the fusion protein MBP–IEGR–*Pgl*PGIP1–6×His–Strep-tag^®^ II. The vector control was generated by eliminating the *pgip* sequence from construct E. *FmPGIII* was PCR amplified from the pGEMT-*FmPGIII* construct and subcloned upstream of the Strep-tag^®^ II in pET22b(+)-Strep-tag^®^ II, allowing the synthesis of the *Fm*PGIII–StrepII fusion protein. DNA manipulations such as agarose gel electrophoresis and bacterial transformation were carried out using standard protocols (Sambrook and Russell, 2001). Restriction digestion (New England Biolabs), plasmid isolation (InnuPREP Plasmid Mini/Midi kit; Analytik Jena Biosciences), ligation (Rapid DNA Ligation kit; Thermo Scientific), and gel extraction (NucleoSpin^®^ Gel and PCR Clean-up kit; Macherey-Nagel) were carried out using kits according to the manufacturer’s instructions.

### *Production and purification of* Pgl*PGIP1 and* Fm*PGIII fusion proteins*

Competent *E. coli* SHuffle^®^ T7 Express [pLysSRARE2] was transformed with the above-described constructs. Expression was carried out in 2 l batch cultures incubated for 24h at 26 °C using auto-induction solutions ‘M’ and ‘5052’ as described by Studier (2005). The total protein was extracted by a freeze–thaw cycle inducing the lysozyme-mediated autolysis of the cells. Additionally, sonication on ice at 40% amplitude, three times for 1min each (10 s on/10 s off cycles) on a Branson Digital Sonifier 250-D (G. Heinemann Ultraschall- und Labortechnik, Germany) was performed. The soluble protein was obtained as supernatant by centrifugation of the cell lysate at 40,000 *g* for 30min at 4 °C.

All purification steps were carried out at 10 °C on a FPLC system (ÄKTAExplorer; GE Healthcare, Freiburg, Germany). A flow rate of 1ml min^–1^ was maintained throughout. The protein peaks were pooled appropriately after each purification step and concentrated using centrifugal concentrators (Vivaspin™20; Sartorius). The intermediate buffer exchanges and desalting steps were carried out using 5ml HiTrap columns (GE Healthcare).

The fusion protein MBP–IEGR–*Pgl*PGIP1–6×His–Strep-tag^®^ II (r*Pgl*PGIP1) was purified in two steps. In the first step, Strep-tag^®^ II-based affinity purification was performed on a 1ml Strep-Tactin Superflow Plus Cartridge (Qiagen) using 50mM NaH_2_PO_4_, 300mM NaCl (pH 8.0) as loading/wash buffer and loading buffer containing 2.5mM d-desthiobiotin (pH 8.0; IBA Lifesciences) as elution buffer according to the manufacturer’s instructions. The desalted eluates were reconstituted in cation-exchange column loading/wash buffer (buffer A: 50mM NaH_2_PO_4_, pH 8.0) and subjected to purification on a 1ml RESOURCE Q Cartridge (GE Healthcare). The matrix-bound proteins were eluted (buffer B: 50mM NaH_2_PO_4_, 1M NaCl, pH 8.0) by applying a stepwise NaCl gradient [0–22% (buffer A to B) in 15min, held for 2min; 22–24% in 5min, held for 2min; 24–28% in 5min, held for 2min; 28–100% in 20 min] to resolve the desired protein from contaminating proteins. The r*Pgl*PGIP1 eluted at 22–24% NaCl held for 5min.

Recombinant *Fm*PGIII-Strep-tag^®^ II (r*Fm*PGIII) and the vector control MBP–IEGR–6×His–Strep-tag^®^ II (rVC) were purified by single-step affinity purification on a Strep-Tactin Superflow Plus Cartridge as described above.

Protein production and purification were monitored by immunoblot analysis of extracted proteins using Strep-Tactin^®^–horseradish peroxidase conjugate as probe (IBA Lifesciences). The chemiluminescence detection of blots was carried out as described previously (Wagenknecht and Meinhardt, 2011). The protein concentration of different samples was determined using a BCA protein assay kit (Pierce) with BSA as the standard.

### PGIP activity assays

*An*PGII (5ng; kind gift from Mr Madhusudhan, University of Mysore, Mysore, India) and r*Fm*PGIII (36ng) were incubated separately in a reaction volume of 200 µl with 0.1mg ml^–1^ of polygalacturonic acid substrate (Sigma) at 30 °C in 50mM sodium acetate buffer (pH 4.2 and 4.6, respectively). PG activity was determined by reducing end-group analysis according to Anthon and Barrett (2002). PGIP activity was assayed by measuring the activity of the PGs pre-incubated with r*Pgl*PGIP1 for 20min at 30 °C. PGIP activity was expressed as the percentage reduction in the number of reducing ends (in µkat mg^–1^ of protein) liberated by PGs in the presence and absence of PGIP. rVC served as the control.

The effect of various parameters such as inhibitor concentration (0.316–12.64nM r*Pgl*PGIP1), substrate concentrations (0.025–0.25mg ml^–1^) and pH (3.5, 4.0, 4.5, and 5.0) on enzyme inhibition was determined. The kinetic parameters were computed by fitting the Michaelis–Menten equation on initial rate experimental data by non-linear fitting using OriginPro7 (Originlab). In separate experiments, the temperature and pH stability of r*Pgl*PGIP1 were studied by pre-incubating them separately for 1h at temperatures ranging from 20 to 100 °C, and for 16h at pH values of 2.0–11.0 at 4 °C, respectively, after which they were reconstituted in the appropriate assay buffer and their inhibition potential was assayed at 30 °C. All experiments were performed twice each in triplicate. The data of a representative experiment was subjected to Tukey’s honestly significant difference (HSD) test following analysis of variance at *P*<0.05.

### *Homology modelling of* Pgl*PGIP1 and* Fm*PGIII*

The initial homology models of *Pgl*PGIP1 and *Fm*PGIII were generated using the MODELLER 9.12 package (Eswar *et al.*, 2006), with the X-ray crystallographic structures of *Pv*PGIP2 [Protein Data Bank identity (PDB ID): 1OGQ] and endo-polygalacturonase I (*Fm*PGI) from *F. moniliforme* isolate FC-10 (PDB ID: 1HG8) serving as templates, respectively. The quality of the generated models was assessed using the Verify3D server (Eisenberg *et al.*, 1997). Post-refinement of structural models was carried out using the KoBa^MIN^ server (Rodrigues *et al.*, 2012), and further geometric accuracy of the constructed models was evaluated using MolProbity 4.02b (Davis *et al.*, 2007). The obtained models were further energy minimized using GROMACS 3.0 (Lindahl and Hess, 2001) with a GROMOS96-53a6 force field to remove geometric inaccuracies and steric clashes with 1000 steepest descent steps terminated at the convergence of maximum force <1000 kJ mol^–1^.

### Electrostatic charge distribution analysis

The adaptive Poisson–Boltzmann Solver program (Unni *et al.*, 2011) was employed to calculate the electrostatic distribution of the following individual proteins and complexes: *Pgl*PGIP1, *An*PGII, *Fm*PGIII, *Pgl*PGIP1–*An*PGII and *Pgl*PGIP1–*Fm*PGIII. PyMOL version 1.2r3pre (PyMOL Molecular Graphics System; Schrödinger, LLC) was used for the visualization of the surface representation.

### Docking and energy minimization

To obtain accurate conformations of *Pgl*PGIP1–*An*PGII and *Pgl*PGIP1–*Fm*PGIII protein complexes, the docking simulation was carried out in two steps; in the first step, the protein pairs were subjected to GRAMM-X (Tovchigrechko and Vakser, 2006) for the initial global search of conformation of the two proteins in complex form and subsequently, such output was resubmitted to the Rosetta 3.4 server (Lyskov and Gray, 2008) for further optimization of docking results based on rigid body orientation and side-chain conformation. The three-dimensional structure of *An*PGII (PDB ID: 1CZF) used in the present docking study was retrieved from the PDB, Research Collaboratory for Structural Bioinformatics.

For energy minimization, molecular dynamics simulations at room temperature were carried out using GROMACS 3.0 (Lindahl and Hess, 2001) with a GROMOS96-53a6 force field for bound *Pgl*PGIP1–*An*PGII and *Pgl*PGIP1–*Fm*PGIII complexes. Protein in complexed form was immersed in a cubic waterbox of space water model with box edges 1 Å from the complexed protein periphery. The overall electric charge of the system was compensated. The system was pre-equilibrated, which involved minimization using the steepest descent method. A molecular dynamics simulation was performed at constant volume and temperature for 1 ns. Snapshots were collected every 2 ps.

### Protein–protein interaction map

Hydrogen bond, ionic, and hydrophobic interactions across protein–protein interfaces were determined by the Protein Interaction Calculator (PIC) server (Tina *et al.*, 2007). Interactions were visualized and analysed using molecular modelling programs PyMOL 1.6 and CHIMERA 1.8 (Pettersen *et al.*, 2004).

### Computational mutagenesis

Computational alanine-scanning mutagenesis was carried out using the Robetta alanine-scanning server (Kim *et al.*, 2004) in order to identify the energetically favourable amino acids at the interface that are important in the stability of the *Pgl*PGIP1–*An*PGII complex. This program scans the protein–protein interface for the ‘hotspots’ and evaluates the changes in the binding free energy of the complex by replacing each of the interface residue with alanine at the binding region.

The relative free energy of binding (ΔΔG_binding_) was calculated as follows:

$$\Delta \Delta {\text{G}}_{\text{binding}}={\text{G}}_{\text{binding}\_\text{wild type}}-{\text{G}}_{\text{binding}\_\text{mutant}}$$

Positive values of ΔΔG_binding_ higher than 1 kcal mol^–1^ indicate that replacement by alanine is predicted to destabilize the complex.

## Results and discussion

### *Isolation of pearl millet* pgip *genes*

Initially, two partial *pgip* sequences: *Pglpgip1p* (407bp, GenBank accession no. GU474543.1) and *Pglpgip2p* (497bp, GenBank accession no. JQ425039), which shared a nucleotide sequence identity of 84%, were isolated from pearl millet using primers based on the consensus sequence of known monocot *pgips*. PGIPs from the same plant, irrespective of whether they are of monocot or dicot origin, share significant identity (Favaron *et al.*, 2000; Ferrari *et al.*, 2003; Janni *et al.*, 2006).

An inverse PCR approach was employed for the isolation of the complete *pgip* coding sequence. For this, a Southern blot analysis was performed to identify appropriate restriction endonucleases for template preparation. About 10 enzymes were tested; however, they did not or only partially cut the genomic pearl millet DNA. Only *Apo*I yielded a continuous digestion pattern and distinct hybridizing fragments of appropriate size (Supplementary Fig. S2 at *JXB* online) and thus was selected to prepare the template. Of the two primer pairs used in the inverse PCR, only InvPGIP1A/InvPGIP1B amplified an intense fragment of ~2.0kb, which was further cloned and sequenced. The sequence assembly resulted in a 2295bp contig (GenBank accession no. JF421287) with an ORF of 1014bp (*Pglpgip1*) (Supplementary Fig. S3 at *JXB* online). The pearl millet coding sequence was found to be uninterrupted, i.e. without any introns, upon comparison of the DNA and cDNA sequences. Most known PGIP-encoding genes have been shown to lack introns; however, exceptions do exist but only in case of dicots such as *Arabidopsis*, peach, raspberry and *Antirrhinum majus* (De Lorenzo *et al.*, 2001).

The Southern blot analysis of pearl millet genomic DNA and the isolation of two PGIP-encoding partial sequences indicated the possible occurrence of *pgip* in pearl millet as a small multigene family. However, further genome organization studies are necessary to confirm this. It has been reported that most plant *pgip* genes exist as small multigene families clustered within a specific chromosomal region (De Lorenzo *et al.*, 2001). *Brassica napus*, in contrast, is the only plant reported so far to contain a large gene family with at least 16 *pgips* (Hegedus *et al.*, 2008).

### *Bioinformatic analysis of* Pglpgip1

**Fig. 1.**
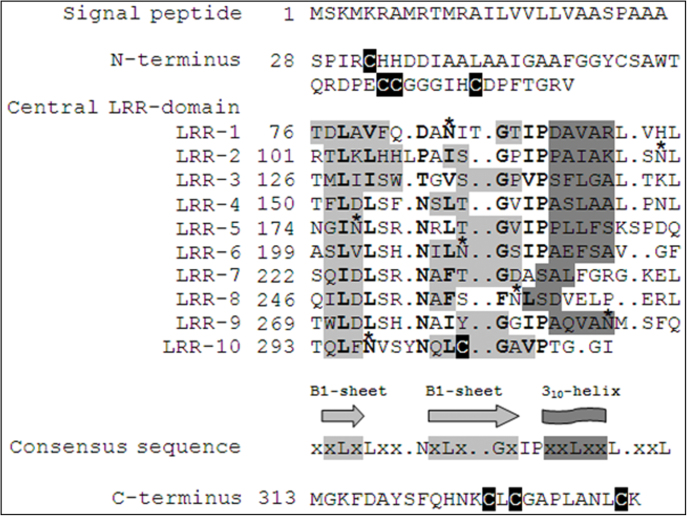
Sequence organization of derived amino acid sequences of *Pgl*PGIP1. The displayed sequence organization is a result of alignment of *Pgl*PGIP1 with *Pv*PGIP2 whose secondary structure has been determined (Di Matteo *et al.*, 2003). The LRR consensus sequence ‘xxLxLxx.NxLx..GxIPxxLxxL.xxL’ is shown. Putative residues contributing to form the secondary structure elements are based on *Pv*PGIP2 and indicated in light grey (sheets B1 and B2) and dark grey (3_10_-helix). Putative glycosylation sites are indicated by an asterisk. The conserved C residues are highlighted in black. Numbering of amino acid residues is shown on the left.

The bioinformatic characterization of *Pglpgip1* was carried out to understand the domain architecture of the deduced protein, its phylogenetic position among known PGIPs, and nature of *cis*-regulatory motifs upstream of the ORF. Only 130 (37%) amino acids were identical between *Pgl*PGIP1and *Pv*PGIP2; however, they were found to have a global alignment score of 95 (Supplementary Fig. S4 at *JXB* online). A putative signal peptide of 27 aa was identified (Fig. 1) for possible apoplastic localization (82% affirmative), which is crucial in plant defence against the invading microbial PGs encountered at the CW (Di Matteo *et al.*, 2003). A central domain was detected in *Pgl*PGIP1, which contained 10 imperfect LRRs of an ~24 aa residue consensus sequence characteristic for PGIPs, xxLxLxx.NxLx..GxIPxxLxxL.xxL (Di Matteo *et al.*, 2003). The pearl millet PGIP also contained two putative β-sheets, B1 and B2, and also the 3_10_-helix similar to *Pv*PGIP2. The central LRR domain has been shown in *Pv*PGIP2 to fold into a right-handed superhelix and is connected to the B1-sheet through loops or β-turns. The B2-sheet is a unique feature not found in other LRR proteins (Di Matteo *et al.*, 2003). In *Pgl*PGIP1, the central domain was flanked by short N- and C- terminal regions with conserved C residues as reported for *Pv*PGIP2, known to be involved in disulfide linkages necessary for the structural integrity of the protein (Di Matteo *et al.*, 2003). Seven putative *N*-glycosylation sites were predicted by NetNGly1.0 with a consensus sequence of N-x-S/T (where x is any amino acid except P), for *N*-glycosylation. Of the seven sites, positions 85 and 297 were conserved in all four rice PGIPs (*Os*PGIP1–4) and both wheat PGIPs reported previously (Janni *et al.*, 2006). N297 was the lone residue to be conserved even in *Pv*PGIP2, whose glycosylation positions have been experimentally mapped (Mattei *et al.*, 2001). N288 was conserved in all rice and wheat PGIPs except in *Os*PGIP1. N258 was conserved in *Os*PGIP2 and *Os*PGIP3, whereas N177 was conserved only in the latter.

**Fig. 2.**
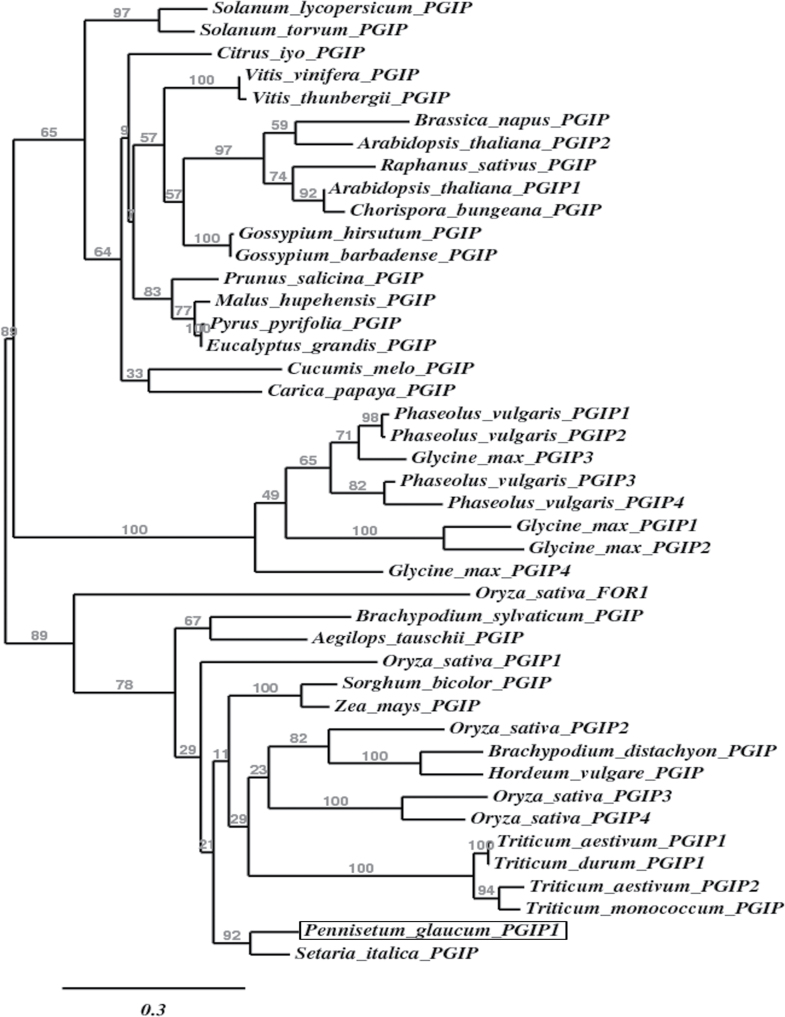
Phylogenetic tree showing the affiliation of *Pgl*PGIP1 among other known monocot and dicot PGIPs. The deduced amino acid sequences of *Pgl*PGIP1 and other known PGIP sequences obtained from GenBank were aligned using MUSCLE version 3.7, curated using the Gblocks version 0.91b, and then submitted to PhyML version 3.0 aLRT for phylogenetic analysis, and the tree was rendered using TreeDyn version 198.3. The position of *Pgl*PGIP1 is highlighted by a box. The branch support values are represented at branch points and the branch length scale is shown below the tree. The protein accession numbers are summarized in Supplementary Table S1.

Phylogenetic analysis of the deduced protein, *Pgl*PGIP1, and other available monocot and dicot PGIP sequences using the maximum-likelihood approach clearly showed monocot and dicot PGIPs forming separate clusters (Fig. 2). *Pgl*PGIP1, was placed among the monocot PGIPs but the millet PGIPs formed a separate branch. *Pgl*PGIP1 was found to share from 50% to just over 60% identity with most monocot PGIPs. The identity with dicots was, however, between 35 and 47%.

Earlier evaluation of *cis*-regulatory elements of various plant *pgip* genes *in silico* detected elements responsive to light, wounding, salicylic acid, abscisic acid, fungal elicitors, ethylene, and various other motifs with unknown functions (Kumar *et al.*, 2009; Lu *et al.*, 2012). The 1078bp sequence upstream of the *Pglpgip1* ORF was analysed *in silico* for the identification of *cis-*elements and showed the presence of numerous important regulatory motifs. The consensus motif sequences and their respective functions retrieved from PlantCARE have been summarized in Supplementary Table S3 at *JXB* online (Supplementary Fig. S5 at *JXB* online shows the position of the *cis*-elements in the upstream sequence). The presence of elements responsive to plant stress hormones (e.g. ACGT-containing abscisic acid response element, TC-rich repeats, TCA element, CGTCA motif, ethylene response elements) and anoxic stress (anaerobic response element) indicates a role for PGIP in pearl millet’s response to biotic and abiotic stress. In bean cultivar ‘Pinto’, *PvPpgip2* was found to be upregulated in response to wounding, oligogalacturonides, glucan derived from *Phytophthora megasperma* f. sp. *glycinea* and salicylic acid (D’Ovidio *et al.*, 2004). A slightly higher accumulation of poplar *Pdpgip4* transcripts was observed over that of *Pdpgip2* transcripts at 1 and 4h after treatment with salicylic acid and H_2_O_2_, respectively (Cheng *et al.*, 2008). However, similar expression levels were reported for both *pgip* genes in case of methyl jasmonate treatment. In Chinese cabbage, *pgip* expression levels were increased in response to abiotic stresses such as water-logging, salt, cold treatment, and mechanical wounding (Ahsan *et al.*, 2005). Significant numbers of elements were found to be responsive to light (e.g. Box 1, G-Box, Specificity protein 1). The effect of light on transcription as well as post-transcriptional processes in plant growth and development has been reported previously (Gilmartin *et al.*, 1990). Plant defence genes have also been proposed to be circadian regulated, and biotic stress responses have been shown to be modulated by a light-sensing network in *Arabidopsis* (Mullineaux *et al.*, 2000; Genoud *et al*., 2002; Wang *et al.*, 2011). A plant’s ability to acclimatize to changes in quality and intensity of light has been found to be critical in mounting an effective response against invading pathogens (Kulheim *et al.*, 2002). Given the presence of numerous light-responsive elements in *Pglpgip1* and other known *pgips*, and the fact that *pgips* are known to be induced by both biotic and abiotic stresses, the relationship between light-induced *pgip* expression and plant defence makes perfect sense.

### *Production of recombinant* Pgl*PGIP1 and* Fm*PGIII*

r*Pgl*PGIP1 was produced in order to assess its potential inhibition against fungal PGs (r*Fm*PGIII and *An*PGII). Our initial attempts to synthesize these proteins in eukaryotic expression systems such as *Nicotiana benthamiana* and *Pichia pastoris* were not successful (data not shown). Furthermore, *Pgl*PGIP1 production in *E. coli* faced protein solubility and purification problems. Hence, several solubility and affinity purification fusion tags were tried out for the soluble production and purification of *Pgl*PGIP1 (data not shown). The N-terminal MBP and C-terminal Strep-tag^®^ II combination of solubility and affinity purification tags, respectively, were found to be optimal for its expression in *E. coli* SHuffle^®^ T7 Express (pLysSRARE2) and efficient purification. *Fm*PGIII expression faced no solubility issues, and hence only a C-terminal Strep-tag^®^ II was incorporated for purification purpose. Expression with pET-22b::MBP-IEGR-*Pgl*PGIP1-6×His-Strep-tag^®^ II in *E. coli* SHuffle^®^ T7 Express (pLysSRARE2) and purification of the resulting recombinant fusion protein by coupling an anion-exchange column downstream of Strep-Tactin Superflow Plus affinity matrix resulted in a homogenous pure protein, r*Pgl*PGIP1, of 79.1kDa (Supplementary Fig. S6A at *JXB* online). The two-step purification resulted in 505- and 1323-fold purification with protein recoveries of 37 and 33%, respectively.

The single-step affinity purification of r*Fm*PGIII and rVC on a Strep-Tactin Superflow Plus Cartridge resulted in relatively homogenous pure proteins of 37.9kDa (Supplementary Fig. S6A) and 46.3kDa (Supplementary Fig. S6B), respectively. Single-step purification of r*Fm*PGIII resulted in 320-fold enrichment with a protein recovery of 72%.

Immunoblot analysis confirmed the synthesis and successful purification of the recombinant proteins (Supplementary Fig. S6). In addition, tandem mass spectrometric analysis of trypsin-digested peptides of purified recombinant proteins was carried out. Subsequent correlation of the resulting spectral data using SEQUEST algorithm (Eng *et al.*, 2008) against a database containing information on theoretical digest of the respective fusion proteins confirmed their identity (data not shown).

### In vitro *inhibition profile of r*Pgl*PGIP1*

**Fig. 3.**
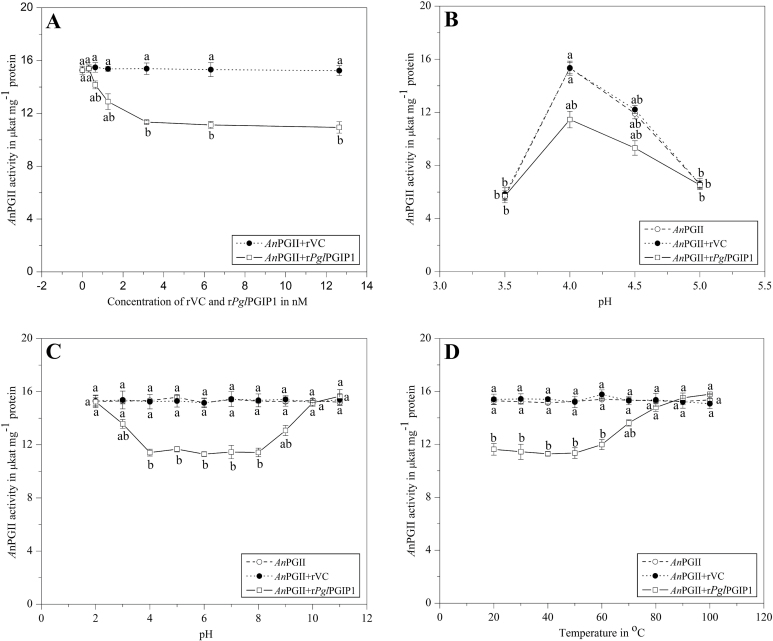
*An*PGII inhibition assay. (A) Effect of inhibitor concentration. *An*PGII (5ng) was assayed with and without inhibitor (r*Pgl*PGIP1/rVC) over a concentration range of 0.316–12.64nM and a graph with the enzyme activity over inhibitor concentration was plotted. (B) pH optimum. *An*PGII (5ng) was assayed with and without inhibitor at a concentration (r*Pgl*PGIP1/rVC) of 3.16nM and a graph with the enzyme activity over pH units was plotted to determine the pH optima of inhibition. (C) pH stability. *An*PGII (5ng) was assayed with and without inhibitor pre-incubated for 16h at pH values of 2.0–10.0 at 4 °C upon reconstitution in the assay buffer [at a concentration (r*Pgl*PGIP1/rVC) of 3.16 nM] and a graph with the enzyme activity over pH units was plotted to determine the pH stability of inhibitor. (D) Temperature stability. *An*PGII (5ng) was assayed with and without inhibitor pre-incubated for 1h at temperatures ranging from 20 to 100 °C [at a concentration (r*Pgl*PGIP1/rVC) of 3.16 nM] and a graph with the enzyme activity over temperature was plotted to determine the temperature stability of inhibitor. The data points are means of a single experiment carried out in triplicates. Results are shown as means±standard error. Means designated with the same letter are not significantly different according to Tukey’s HSD test at *P*<0.05.

The *in vitro* inhibition of r*Pgl*PGIP1 against *An*PGII and r*Fm*PGIII was carried out in order to compare it with the known inhibition profile of *Pv*PGIP2 against the same two PGs. Intact MBP–*Pgl*PGIP1 fusion protein was employed in the inhibition studies as protease factor Xa digestion for cleaving off MBP resulted in random degradation of protein (data not shown). r*Pgl*PGIP1 displayed a differential activity profile against the fungal PGs used in the present study, with only a partial inhibition observed against *An*PGII (Fig. 3A) and no inhibition against r*Fm*PGIII. Hence, the effect of various parameters on *An*PGII inhibition by r*Pgl*PGIP1 has been presented below. The rVC, as expected, showed no inhibition of either enzyme. Possibly, the lack of glycosylation of r*Pgl*PGIP1 produced in *E. coli* could be a factor responsible for the limited inhibition observed.

*Pv*PGIP2 has been reported to competitively inhibit *Fm*PGI by binding at its substrate-binding site (Leckie *et al.*, 1999), whereas *Fm*PGIII, which shares 91.7% amino acid identity with *Fm*PGI, evaded inhibition by the same PGIP (Sella *et al.*, 2004). Although a structural explanation is lacking, an attempt was made to explain the observed effect using amino acid sequence alignment. The chief difference between the *Fm*PGIII and *Fm*PGI consist of one short stretch of substitutions in the N-terminal region and ﬁve substitutions, two of which are close to the active site. These polymorphic sites may have led to subtle changes in the protein structure, thus preventing the accessibility of the substrate-binding site of *Fm*PGIII for interaction with *Pv*PGIP2 (Sella *et al.*, 2004). As mentioned earlier, although sharing 37% amino acid identity, the global alignment score of *Pgl*PGIP1 and *Pv*PGIP2 is very high due to the degeneracy of residues in the LRR domain. Hence, lack of potency of *Pgl*PGIP1 against *Fm*PGIII is understandable. Assessment of the *Pgl*PGIP1–*Fm*PGI interaction could further be useful for a deeper comparative profiling of inhibition with *Pv*PGIP2.

### *Effect of inhibitor concentration on* An*PGII activity*

No inhibition was observed at the lowest r*Pgl*PGIP1 concentration tested, but a gradual increase in inhibition was observed with increasing inhibitor concentration (Fig. 3A). A significant increase in inhibition was observed at r*Pgl*PGIP1 concentrations of 0.632, 1.26, and 3.16nM with inhibition being 7, 16, and 26%, respectively. However, a further increase of the r*Pgl*PGIP1 concentration (up to 6.32 and 12.64nM) resulted in marginally increased inhibition reaching a value of 28% at most. PGIP concentrations of 3.16nM and/or 1.26nM were used in further studies.

Although the pattern of inhibition of both r*Pgl*PGIP1 and *Pv*PGIP2 against the same two PGs was similar, bean PGIP showed complete inhibition against *An*PGII at concentrations lower than that used in the present study (Leckie *et al.*, 1999; Sella *et al.*, 2004). As observed in the present study, such partial inhibitions of various PGs have been reported in several PG/PGIP systems (Gomathi and Gnanamanickam, 2004). PGIPs from cotton showed 50 and 25% inhibition against PGs from *Aspergillus niger* and *Verticillium dahlia*, respectively (James and Dubery, 2006). Wheat PGIP, on the other hand, showed just over 5% inhibition of PGs from *A. niger* and *F. moniliforme* (Kemp *et al.*, 2003). PGIPs are very diverse in their inhibition specificity and potential, i.e. PGIPs isolated from a single plant can inhibit different PGs, the inhibitors isolated from different plants have been found to inhibit the same PG, some PGIPs have the ability to inhibit a multitude of PGs, and the degree of inhibition has also been found to vary greatly (Gomathi and Gnanamanickam, 2004). PGIPs have also been noted to have the potential to inhibit *in vitro* the PGs of those microbes that are non-pathogenic or to which they are generally not exposed, as in our case (Gomathi and Gnanamanickam, 2004). The presence of PGIPs with different inhibition potentials could be a ploy employed by the host ensuring the accumulation of elicitor-active oligogalacturonides.

### *Mode of* An*PGII inhibition*

The control protein, rVC (1.26 and 3.16nM) had no effect on the kinetic parameters of *An*PGII (Table 1). r*Pgl*PGIP1 (1.26 and 3.16nM), however, was found to decrease the *V*_max_ without affecting the *K*_m_ of *An*PGII. Hence, the mode of inhibition was established to be non-competitive in nature. PGIPs depending on the PG are known to employ competitive, non-competitive, and mixed modes of inhibition, thus indicating that PGIPs are capable of recognizing different structural motifs in its various protein partners (Sicilia *et al.*, 2005). PGIPs from different sources have been shown to display similar as well as different inhibition kinetics against the same enzyme. For example, *Bc*PG from *Botrytis cineria* was competitively inhibited by pear PGIP but in a mixed mode by the inhibitor from bean (Abu-Goukh and Labavitch, 1983; Sicilia *et al.*, 2005). However, *An*PGII was non-competitively inhibited by PGIPs of tomato and bean (Stotz *et al.*, 2000), as in our case.

**Table 1.** The kinetic parameters of AnPGII with and without rVC and rPglPGIP1 *An*PGII (5ng) was assayed using a substrate concentration range of 0.025–0.25mg ml^–1^ with and without inhibitors (r*Pgl*PGIP1/rVC) at concentrations of 1.26 and 3.16nM. The kinetic parameters were calculated by fitting the Michaelis–Menten equation on initial rate experimental data by non-linear fitting using OriginPro 7 (Originlab).

	*K*_*m*_ (mg ml^–1^)	*V*_*max*_ (μkat mg^–1^ protein)
*An*PGII	0.091	28.5
*An*PGII+rVC (1.26nM)	0.091	28.5
*An*PGII+rVC (3.16nM)	0.092	28.6
*An*PGII+r*Pgl*PGIP1 (1.26nM)	0.092	24.3
*An*PGII+r*Pgl*PGIP1 (3.16nM)	0.091	21.3

### *pH optima of* An*PGII inhibition*

The *An*PGII inhibition by r*Pgl*PGIP1 (3.16nM) over a pH range of 3.5–5.0 showed the pH optima for inhibition to be between 4.0 and 4.5 with 25 and 22% inhibition, respectively (Fig. 3B). However, no inhibition was observed at pH 3.5 and 5.0. The pH optima for inhibition of *A. niger* PG by PGIP-I and PGIP-II of guava fruit was determined to be 4.2, whereas that for PGIP-III was 4.4 when assayed over a pH range of 4.0–5.5 (Deo and Shastri, 2003). A study involving interaction of *Pv*PGIP2 with five isoforms of *A. niger* PGs (*An*PGI, *An*PGII, *An*PGA, *An*PGB, and *An*PGC) showed *Pv*PGIP2 to greatly reduce the activity of three of the PG isoforms—*An*PGI, *An*PGII and *An*PGB—at all tested pH values. For *An*PGA and *An*PGC, *Pv*PGIP2 displayed inhibition only at pH 4.75 and below pH 4.2, respectively. At pH 5.0, however, *Pv*PGIP2 was found to activate the two PG isoforms (Kemp *et al.*, 2004). This diversity of PGIP activity in different pH environments is crucial in countering the multitude of PGs encountered from various pathogens with different pH optima. In addition, such differential PGIP activity has been proposed to be involved in mounting a better host defence response through the generation of a steady-state concentration of biologically active oligogalacturonides (Kemp *et al.*, 2004).

### *pH and thermal stability of r*Pgl*PGIP1*

r*Pgl*PGIP1 (at 3.16nM) was found to be stable over a wide range of pH values from 4.0 to 8.0 (Fig. 3C). The inhibition potential decreased by approximately 50% at pH 3.0 and 9.0, with no observed inhibition at other tested pH values. Guava PGIPs were reported to be stable only over a narrow pH range of 2.0–4.0 (Deo and Shastri, 2003), but the PGIP from chilli retained >50% activity at pH 3.0 and 8.0 (Shivashankar *et al.*, 2010).

r*Pgl*PGIP1 (at 3.16nM) was found to be equally active from 20 to 50 °C (Fig. 3D). The activity dropped very slightly to 22% at 60 °C and significantly at 70 and 80 °C to 11 and 3%, respectively. No inhibition was observed beyond this temperature. Earlier studies on thermal tolerance of the protein have shown it to be relatively thermostable. PGIPs from ‘Bartlett’ pear (Abu-Goukh *et al.*, 1983) and orange (Barmore and Nguyen, 1985) retained significant activity at 60 °C. The latest study on PGIP isolated from tomato was found to retain partial inhibitory activity, even at 100 °C (Schacht *et al.*, 2011).

### In silico *analysis of* Pgl*PGIP1*–An*PGII and* Pgl*PGIP1*–Fm*PGIII complexes*

*In silico* protein modelling, docking, and computational mutagenesis studies were carried out to account for the differential behaviour of pearl millet PGIP against *An*PGII and *Fm*PGIII, and for the limited similarity in the inhibition of *Pgl*PGIP1 and *Pv*PGIP2 against the same two PGs. It was necessary to predict the underlying structural basis of interaction as the two PGIPs share an amino acid identity of just 37%.

### *Homology modelling of* Pgl*PGIP1* and Fm*PGIII*

The initial homology models of *Pgl*PGIP1 (Supplementary Fig. S7A at *JXB* online) and *Fm*PGIII (Supplementary Fig. S7B) were generated using already known structures of *Pv*PGIP2 (PDB ID: 1OGQ) and *Fm*PGI (PDB ID: 1HG8) serving as templates, respectively. The quality of the models was assessed using Verify3D. Scores predicted for each residue of our constructed model were >0.1, indicating that all of the residues were located in favourable structural environments. After post-refinement with the KoBa^MIN^ program, the final models were revalidated using MolProbity. In the present study, the MolProbity score for both the models was >85%, representing a good quality model. Summarized results from KoBa^MIN^ and MolProbity are given in Supplementary Table S4 at *JXB* online. Models further energy minimized using GROMOS96-53a6 force field to remove any local strains were finally used for the docking studies.

### *Docking studies of* Pgl*PGIP1*–An*PGII and* Pgl*PGIP1*–Fm*PGIII complexes*

**Fig. 4.**
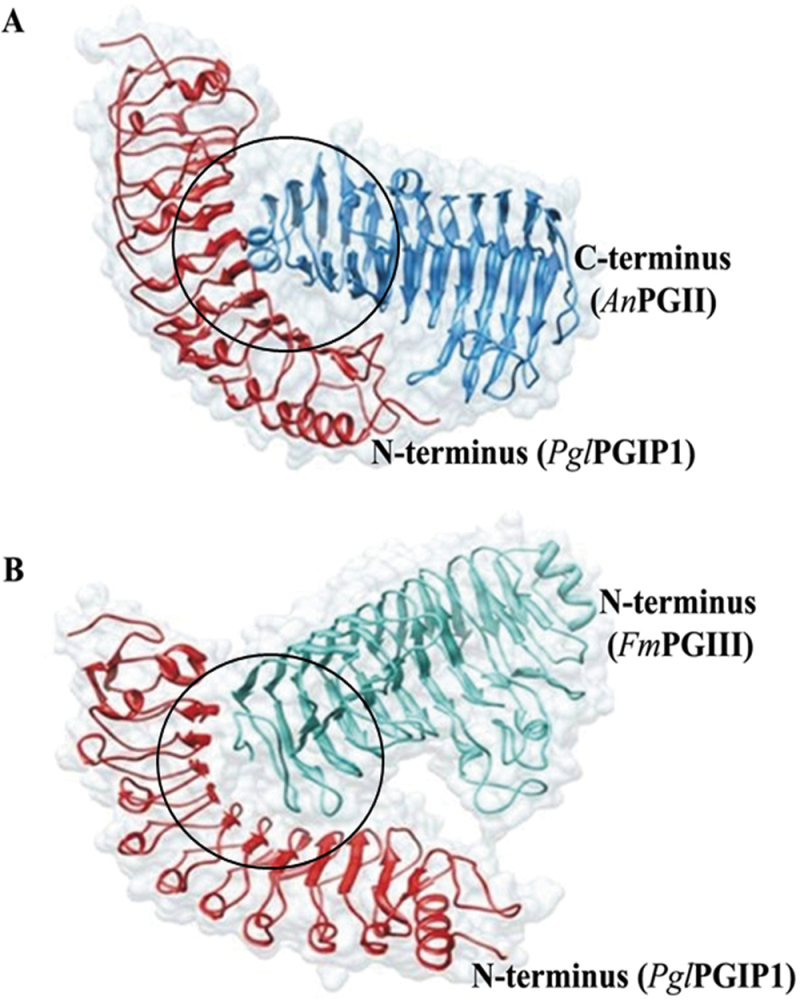
Protein docking analysis. Docked poses of *Pgl*PGIP1–*An*PGII (A) and *Pgl*PGIP1–*Fm*PGIII (B) complexes. *Pgl*PGIP1 interacts through its solvent-exposed concave cavity with *An*PGII and *Fm*PGIII at their N- and C-termini (circled in black), respectively. The substrate-binding site in *Fm*PGIII appears to be more exposed compared with that of *An*PGII. (This figure is available in colour at *JXB* online.)

To predict the conformation and the putative interactions between *Pgl*PGIP1 with *An*PGII and *Fm*PGIII, two different protein–protein docking programs, GRAMM-X and Rosetta 3.4, were used. Firstly, using GRAMM-X, fast-Fourier-transformation-based unrestrained rigid body docking was performed, which generated the top 10 solutions of the complexes. In the absence of the detailed data on the binding mode or mutational studies on *Pgl*PGIP1, we selected the docked complex based on the present *in vitro* inhibition studies, as well as available experimental evidence from *Pv*PGIP2 interactions with the two enzymes (Leckie *et al.*, 1999; Federici *et al.*, 2001; King *et al.*, 2002; Di Matteo *et al.*, 2003; Sella *et al.*, 2004; Maulik *et al.*, 2009; Benedetti *et al.*, 2011). For further optimization, selected models from GRAMM-X were subjected to Rosetta 3.4. This docking algorithm searches a set of conformations from a given starting conformation for the optimal fit between the two partners. It employs a Monte Carlo search followed by simultaneous optimization of side-chain conformations. The resulting ‘decoys’ obtained from the docking simulations were ranked using an energy function dominated by van der Waals interactions, an implicit solvation model and an orientation-dependent hydrogen bonding potential. Selection of the best docked conformation obtained from Rosetta 3.4 was based on: (i) docking score being an overall measure of the energy of the complex; (ii) an interface score representing the score of the complex minus the total score of each partner in isolation; and (iii) involvement of any residues in the interaction evident from the experimental studies from *Pv*PGIP2. The best docked conformations of the *Pgl*PGIP1–*An*PGII and *Pgl*PGIP1–*Fm*PGIII complexes are shown in Fig. 4, and the docking and interface scores are presented in Supplementary Table S5 at *JXB* online.

From the docked protein complexes, it was suggested that *An*PGII (Fig. 4A) and *Fm*PGIII (Fig. 4B) both interact with *Pgl*PGIP1 at its concave surface, but their binding orientations differ. The residues at the β-strand/β-turn motif of the central LRR domain constitute the solvent-exposed concave surface of the PGIP and this region determines the binding specificity for PGs (Kobe and Deisenhofer, 1994). LRRs are known to be versatile protein recognition domains present in over 14,000 proteins (Matsushima and Miyashita, 2012). In case of the *Pgl*PGIP1–*An*PGII complex, the concave site of *Pgl*PGIP1 interacts with the N-terminal site of *An*PGII, whereas in the *Pgl*PGIP1–*Fm*PGIII complex, the concave site of *Pgl*PGIP1 interacts with the C-terminal site of *Fm*PGIII. The substrate-binding site in *Fm*PGIII appeared to be more exposed compared with that of *An*PGII. In terms of the docking score, binding of *Pgl*PGIP1 with *An*PGII was predicted to be stronger in comparison with *Fm*PGIII. These *in silico* results are consistent with the *in vitro* outcomes. Interaction of *Pv*PGIP2 with *An*PGII and *Fm*PGI showed some residues important for interaction with one PG to be dispensable for interaction with the other, suggesting that different but overlapping subsets of residues are vital in binding different ligands (Leckie *et al.*, 1999). The molecular docking simulation of the *Bc*PG1–*Pv*PGIP2 complex showed the B1-sheet of *Pv*PGIP2 to interact with the N-terminus of *Bc*PG1, and the active site of *Bc*PG1 was partially buried by the *Pv*PGIP2 C-terminus (Sicilia *et al.*, 2005). Analysis of the *Pv*PGIP2–*Fm*PG1 (now referred to as *Fp*PG) complex showed the residues present at both the convex and concave side of the PGIP N-terminus to be involved in interaction with the loops surrounding the active site of the PG (Benedetti *et al.*, 2011). These studies further demonstrated the structural flexibility and the versatility of PGIP binding interactions with the various PGs.

### *Evaluation of protein–protein interactions in* Pgl*PGIP1*–An*PGII and* Pgl*PGIP1*–Fm*PGIII complexes by PIC analysis*

Energy-minimized *Pgl*PGIP1–*An*PGII and *Pgl*PGIP1–*Fm*PGIII complexes were analysed using the PIC server to predict all possible types of interactions at the protein–protein interfaces, and the putative amino acid residues involved were mapped (Table 2). It was apparent from the data that the protein–protein contacts in both complexes were mediated through all types of interactions, i.e. ionic, hydrophobic, and hydrogen bonds, whereas the ionic and hydrophobic interactions were predominating at the surface. However, in the docked as well as energy-minimized structures, the *Pgl*PGIP1–*An*PGII complex was found to have a stronger binding interaction than that recorded in the *Pgl*PGIP1–*Fm*PGIII complex.

**Table 2.** Protein interaction analysis of PglPGIP1–AnPGII and PglPGIP1–FmPGIII complexes using the PIC The residue pairs involved in the interacting complexes, sorted according to the type of interaction, are shown (the PGIP residue numbering followed excludes the putative signal peptide).

Hydrophobic interactions			
In *Pgl*PGIP1–*An*PGII complex		In *Pgl*PGIP1–*Fm*PGIII complex	
*Pgl*PGIP1	*An*PGII	*Pgl*PGIP1	*Fm*PGIII
F54	Y130	W105	A306
M100	W85	W243	I332
I102	W85	L268	A330
W105	A43		
F124	P56		
F129	A40		
A172	P56		
V175	A36		
Side-chain H-bonding interactions			
T28	S234	R153	N266
H79	T64	D222	T332
N145, N147	E83		
S195	E54		
Q219	E54		
Ionic interactions			
D31	R233	D56	K269
D42	K124	D290	K300
D50	K127		
R74	E83, E84		
L77	D62		
H79	D62		
D126	K39		
R240	E54		

### *Electrostatic surface charge distribution on* Pgl*PGIP1*, An*PGII, and* Fm*PGIII individually, and the* Pgl*PGIP1*–An*PGII and* Pgl*PGIP1*–Fm*PGIII complexes*

**Fig. 5.**
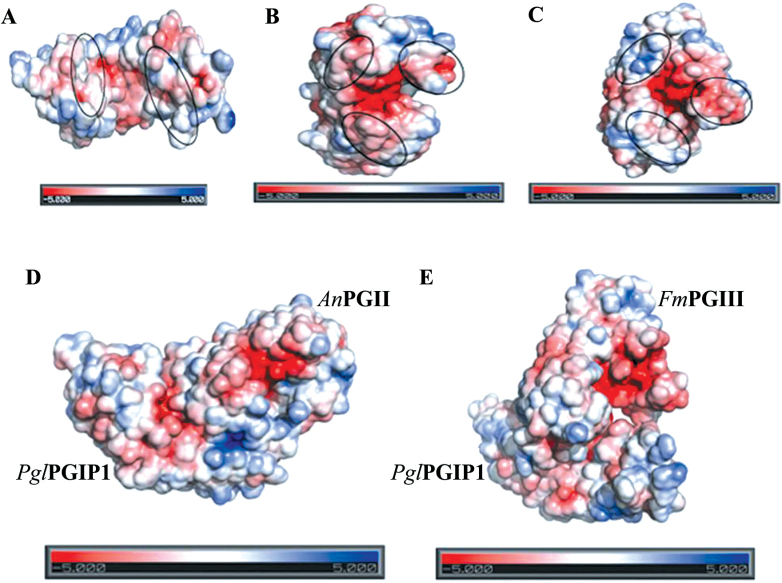
Electrostatic surface potential of individual proteins and protein complexes. Electrostatic potential maps of *Pgl*PGIP1 (A), *An*PGII (B), *Fm*PGIII (C), *Pgl*PGIP1–*An*PGII (D), and *Pgl*PGIP1–*Fm*PGIII (E) complexes on which surface colours are fixed at red (–5) or blue (+5). Marked regions display the difference in charge distributions in surface maps of the individual proteins.

To illustrate the charge distributions of molecules, a three-dimensional electrostatic surface potential was generated separately on *Pgl*PGIP1 (Fig. 5A), *An*PGII (Fig. 5B), and *Fm*PGIII (Fig. 5C), as well as on the *Pgl*PGIP1–*An*PGII (Fig. 5D) and *Pgl*PGIP1–*Fm*PGIII (Fig. 5E) complexes.

The surface comparison of *An*PGII and *Fm*PGIII suggested that the structures not only differed with respect to charge distribution but also differed in shape. This difference in the surface potential of the individual enzymes was also reflected in their predicted differential binding interactions with *Pgl*PGIP1. Once again, the *Pgl*PGIP1–*An*PGII complex was more compact than *Pgl*PGIP1–*Fm*PGIII, with the active site cleft of *Fm*PGIII exposed to a greater extent, which further explicated the *in vitro* results. This is consistent with an earlier report that electrostatic and van der Waals interactions play a significant role in the proper recognition and discrimination of PGs by PGIPs (Maulik *et al.*, 2009).

### *Analysis of* Pgl*PGIP1*–An*PGII interaction by computational alanine-scanning mutagenesis*

The residues at the β-strand/β-turn motif of *Pv*PGIP2 have been shown to interact with *An*PGII at the D110 α-helix, opposite the substrate-binding site, which fits perfectly with the non-competitive mode of inhibition (Stotz *et al.*, 2000). To identify the hotspot residues involved in the interaction of *Pgl*PGIP1 and *An*PGII, which also follows the non-competitive mode of inhibition, a computative alanine mutagenesis of residues at the interface was carried out. Virtual scanning was performed over all interface residues and changes in the binding free energy were calculated upon alanine substitution of residues at protein–protein interfaces.

**Fig. 6.**
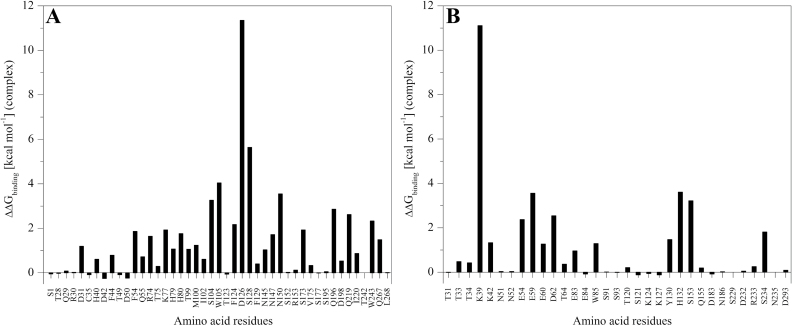
Computational alanine mutagenesis of *Pgl*PGIP1–*An*PGII interface residues. The plot displays the contribution of individual interacting residues from *Pgl*PGIP1 (the PGIP residue numbering followed excludes the putative signal peptide) (A) and *An*PGII (B) in the stability of the *Pgl*PGIP1–*An*PGII complex. Interface residues were defined as those residues with a side chain having at least one atom within a sphere with 4 Å radius of an atom belonging to the other partner in the complex and binding hotspots defined as those residues that show ∆∆G_binding_ >1 kcal mol^–1^.

From the existing experimental data on *Pv*PGIP2, the amino acids H104, Y105, Y107, D131, V152, F201, Q224, and K225 have been reported to be important in interaction with *An*PGII (Leckie *et al.*, 1999; Casasoli *et al.*, 2009; Spinelli *et al.*, 2009; Benedetti *et al.*, 2011). Alignment of *Pv*PGIP2 and *Pgl*PGIP1 sequences identified *Pgl*PGIP1 residues T99, M100, I102, D126, N147, Q196, Q219, and I220, respectively, at positions corresponding to the *Pv*PGIP2 residues mentioned above. Interestingly, computational mutagenesis predicted six of them to be hotspot residues significantly involved in interaction, except for I102 and I220, with ΔΔG_binding_ values of 0.61 and 0.87 kcal mol^–1^, respectively (Fig. 6A). In addition, all the identified residues could be pinned down to the β-strand/β-turn solvent-exposed region except for D31, N145, and R240 found localized at the N-terminus, LRR-4 and LRR-8 in the central domain (Fig. 1), respectively.

The information on *An*PGII residues involved in interaction with PGIPs is very limited. Studies on conformational changes in *An*PGII–HG–*Pv*PGIP2 using amide-exchange mass spectrometry identified four residues (E95, G104, D110, and I139) to be involved in *Pv*PGIP2 binding (King *et al.*, 2002). The residues were found to lie opposite the substrate-binding site around the underside of the barrel near the D110 α-helix, consistent with the reported non-competitive mode of inhibition. However, the docking pose and protein–protein interaction analysis as well as the computational alanine-scanning mutagenesis of the *Pgl*PGIP1–*An*PGII complex (Fig. 6A, B) identified that the N-terminal region of *An*PGII interacts with the concave surface of *Pgl*PGIP1. Many of the identified residues were found to be localized mainly at the small α-helix, and β-strands of β-sheet PB1 at the N-terminus of *An*PGII away from the substrate-binding surface, which again is consistent with the observed non-competitive mode of inhibition.

Most residues of *Pgl*PGIP1 and *An*PGII identified by protein–protein interaction analysis as involved in interaction were also identified as significantly important upon computational alanine-scanning mutagenesis (Table 2 and Fig. 6). The D126 (*Pgl*PGIP1)–K39 (*An*PGII) interaction was predicted by alanine mutation studies to be the most significant binding contact in the *Pgl*PGIP1–*An*PGII complex.

The above results predicted the preferential use of very similar motif regions in PG recognition by the two PGIPs with few identical amino acids. *Pv*PGIP2 shares 99 and 88% identity with *Pv*PGIP1 and *Gm*PGIP3, respectively, at the amino acid level. However, assessment of their inhibition against *An*PGII and *Fm*PGI, unexpectedly showed *Pv*PGIP2 and *Gm*PGIP3 to share similar inhibition profiles, but *Pv*PGIP2 and *Pv*PGIP1 did not (Leckie *et al.*, 1999; D’Ovidio *et al.*, 2006). Docking studies of these complexes proposed that not just sequence similarity but also conservation of key structural features are crucial in preserving the function mediated by appropriate protein-–protein interactions (Maulik *et al.*, 2009). Even though *Pgl*PGIP1 and *Pv*PGIP2 employed similar motifs for interaction with *An*PGII at non-substrate-binding sites, they engaged different regions of *An*PGII. Subtle differences in the overlapping residues as well as recruitment of additional, completely different amino acids could be responsible for the observed binding disparity.

*In silico* alanine mutation studies predicted D126 (*Pgl*PGIP1)–K39 (*An*PGII) interaction as the single most important binding contact in the *Pgl*PGIP1–*An*PGII complex. This could be important as the D126 position is an evolutionarily conserved residue in PGIPs. The importance of a single amino acid in protein–protein interaction has been observed earlier. *Pv*PGIP1 gained the ability to inhibit *Fm*PGI through a single amino acid substitution of K224 into the corresponding amino acid of *Pv*PGIP2, a Q (Leckie *et al.*, 1999). Hence, *in silico* analysis of the *Pgl*PGIP1–*An*PGII complex is a good starting point for further experimental mutational analysis to arrive at the actual residues involved in protein–protein interactions.

In conclusion, the present study, together with earlier literature, suggests that the PG–PGIP interactions are complex, and structural and mutational analyses of various PG–PGIP complexes would be needed before a comprehensive generalized conclusion could be drawn about structure–function correlation. Pearl millet is afflicted by many fungal and bacterial diseases. Downy mildew disease alone accounts for annual production losses in the range of 20–40% (Singh, 1995). Such protein–protein interaction studies will be crucial from the perspective of generation of designer host proteins with improved combat potential against the ever-evolving pathogen virulence factors.

## Supplementary data

Supplementary data are available at *JXB* online.

Supplementary Table S1. List of plant PGIPs used in the protein phylogenetic analysis.

Supplementary Table S2. Construction of *Pgl*PGIP1, vector control and *Fm*PGIII expression plasmids for expression in *Escherichia coli* SHuffle^®^ T7 Express (pLysSRARE2).

Supplementary Table S3. Upstream sequence analysis of the *Pglpgip1* gene.

Supplementary Table S4. Refinement of *Pgl*PGIP1 and *Fm*PGIII models.

Supplementary Table S5. Docking and interface scores of *Pgl*PGIP1–*An*PGII and *Pgl*PGIP1–*Fm*PGIII complexes.

Supplementary Fig. S1. Pictorial representation of the *Pgl*PGIP1, vector control and *Fm*PGIII expression plasmids for expression in *Escherichia coli* SHuffle^®^ T7 Express (pLysSRARE2).

Supplementary Fig. S2. Southern blot analysis of pearl millet total DNA.

Supplementary Fig. S3. Nucleotide and derived amino acid sequences of the pearl millet *Pglpgip1* gene.

Supplementary Fig. S4. Alignment of *Pgl*PGIP1 and *Pv*PGIP2 sequences using the T-Coffee multi-alignment tool.

Supplementary Fig. S5. Upstream *cis*-regulatory elements in the *Pglpgip1* gene.

Supplementary Fig. S6. Purification of recombinant *Pgl*PGIP1, vector control and *Fm*PGIII fusion proteins synthesized in *Escherichia coli* SHuffle^®^ T7 Express (pLysSRARE2).

Supplementary Fig. S7. Homology modelling of *Pgl*PGIP1 and *Fm*PGIII.

Supplementary Data

## Abbreviations:

6×His: hexa-histidine tag
CWs: cell walls
HG: homogalacturonan
HSD: honestly significant difference
LRR: leucine-rich repeat
MBP: maltose-binding protein
ORF: open reading frame
PDB ID: Protein data bank identity
PGIPs: polygalacturonase-inhibitor proteins
PGs: polygalacturonases
PIC: Protein Interaction Calculator
r: recombinant
rVC: vector control protein.

## References

[CIT0001] Abu-Goukh AA Greve LC Labavitch JM 1983 Purification and partial characterization of “Barlett” pear fruit polygalacturonase inhibitors. Physiological Plant Pathology 23, 111–122

[CIT0002] Abu-Goukh AA Labavitch JM 1983 The *in vivo* role of “Bartlett” pear fruit polygalacturonase inhibitors. Physiological Plant Pathology 23, 123–135

[CIT0003] Ahsan N Yoon HS Jo J 2005 Molecular cloning of a *Bc*PGIP cDNA from *Brassica campestris* and its expression to several stresses. Plant Science 169, 1081–1089

[CIT0004] Anisimova M Gascuel O 2006 Approximate likelihood-ratio test for branches: a fast, accurate, and powerful alternative. Systematic Biology 55, 539–552 16785212 10.1080/10635150600755453

[CIT0005] Anthon GE Barrett DM 2002 Determination of reducing sugars with 3-methyl-2-benzothiazolinonehydrazone. Analytical Biochemistry 305, 287–289 12054461 10.1006/abio.2002.5644

[CIT0006] Barmore CR Nguyen TK 1985 Polygalacturonase inhibition in rind of “Valencia” orange infected with *Diplodia natalensis* . Phytopathology 75, 446–449

[CIT0007] Benedetti M Leggio C Federici L De Lorenzo G Pavel NV Cervone F 2011 Structural resolution of the complex between a fungal polygalacturonase and a plant polygalacturonase-inhibiting protein by small-angle X-ray scattering. Plant Physiology 157, 599–607 21859985 10.1104/pp.111.181057 PMC3192570

[CIT0008] Bonivento D Pontiggia D Matteo AD Fernandez-Recio J Salvi G Tsernoglou D Cervone F De Lorenzo G Federici L 2008 Crystal structure of the endopolygalacturonase from the phytopathogenic fungus *Colletotrichum lupini* and its interaction with polygalacturonase-inhibiting proteins. Proteins: Structure, Function, and Bioinformatics 70, 294–299 10.1002/prot.21610 17876815

[CIT0009] Casasoli M Federici L Spinelli F Di Matteo A Vella N Scaloni F Fernandez-Recio J Cervone F De Lorenzo G 2009 Integration of evolutionary and desolvation energy analysis identifies functional sites in a plant immunity protein. Proceedings of the National Academy of Sciences, USA 106, 7666–7671 10.1073/pnas.0812625106 PMC2678593 19372373

[CIT0010] Cheng Q Cao Y Pan H Wang M Huang M 2008 Isolation and characterization of two genes encoding polygalacturonase-inhibiting protein from *Populus deltoids* . Journal of Genetics and Genomics 35, 631–638 18937920 10.1016/S1673-8527(08)60084-3

[CIT0011] Chevenet F Brun C Bañuls A Jacq B Christen R 2006 TreeDyn: towards dynamic graphics and annotations for analyses of trees. BMC Bioinformatics 9, 1–9 10.1186/1471-2105-7-439 PMC1615880 17032440

[CIT0012] D’Ovidio R Raiola A Capodicasa C Devoto A Pontiggia D Roberti S Galletti R Conti E O’Sullivan D De Lorenzo G 2004 Characterization of the complex locus of bean encoding polygalacturonase-inhibiting proteins reveals subfunctionalization for defense against fungi and insects. Plant Physiology 135, 2424–2435 15299124 10.1104/pp.104.044644 PMC520809

[CIT0013] D’Ovidio R Roberti S Di Giovanni M Capodicasa C Melaragni M Sella L Tosi P Favaron F 2006 The characterization of the soybean polygalacturonase-inhibiting proteins (*Pgip*) gene family reveals that a single member is responsible for the activity detected in soybean tissues. Planta 224, 633–645 16501991 10.1007/s00425-006-0235-y

[CIT0014] Davis IW Leaver-Fay A Chen VB 2007 MolProbity: all-atom contacts and structure validation for proteins and nucleic acids. Nucleic Acids Research 35, W375–W383 17452350 10.1093/nar/gkm216 PMC1933162

[CIT0015] De Lorenzo G D’Ovidio R Cervone F 2001 The role of polygalacturonase-inhibiting proteins (PGIPs) in defense against pathogenic fungi. Annual Review of Phytopathology 39, 313–335 10.1146/annurev.phyto.39.1.313 11701868

[CIT0016] Deo A Shastri N 2003 Purification and characterization of polygalacturonase-inhibitory proteins from *Psidium guajava* Linn. (guava) fruit. Plant Science 164, 147–156

[CIT0017] Dereeper A Guignon V Blanc G Audic S Buffet S Chevenet F Guindon S Lefort V Lescot M Gascuel O 2008 Phylogeny.fr: robust phylogenetic analysis for the non-specialist. Nucleic Acids Research 36, 465–469 10.1093/nar/gkn180 PMC2447785 18424797

[CIT0018] Di Matteo A Federici L Mattei B Salvi G Johnson KA Savino C De Lorenzo G Tsernoglou D Cervone F 2003 The crystal structure of polygalacturonase-inhibiting protein (PGIP), a leucine-rich repeat protein involved in plant defense. Proceedings of the National Academy of Sciences, USA 100, 10124–10128 10.1073/pnas.1733690100 PMC187787 12904578

[CIT0019] Edgar RC Drive RM Valley M 2004 MUSCLE: multiple sequence alignment with high accuracy and high throughput. Nucleic Acids Research 32, 1792–1797 15034147 10.1093/nar/gkh340 PMC390337

[CIT0020] Eisenberg D Lüthy R Bowie JU 1997 VERIFY3D: Assessment of protein models with three-dimensional profiles. Methods in Enzymology 277, 396–404 9379925 10.1016/s0076-6879(97)77022-8

[CIT0021] Eng JK Fischer B Grossmann J MacCoss MJ 2008 A Fast SEQUEST cross correlation algorithm. Journal of Proteome Research 7, 4598–4602 18774840 10.1021/pr800420s

[CIT0022] Eswar N Webb B Marti-Renom MA Madhusudhan MS Eramian D Shen M Pieper U Sali A 2006 Comparative protein structure modeling using modeller. Current Protocols in Bioinformatics 15, 5.6.1–5.6.30 10.1002/0471250953.bi0506s15 PMC4186674 18428767

[CIT0023] Farina A Rocchi V Janni M Benedettelli S De Lorenzo G D’Ovidio R 2009 The bean polygalacturonase-inhibiting protein 2 (*Pv*PGIP2) is highly conserved in common bean (*Phaseolus vulgaris* L.) germplasm and related species. Theoretical and Applied Genetics 118, 1371–1379 19238348 10.1007/s00122-009-0987-4

[CIT0024] Favaron F Destro T D’Ovidio R 2000 Transcript accumulation of polygalacturonase inhibiting protein (PGIP) following pathogen infections in soybean. Journal of Plant Pathology 82, 103–109

[CIT0025] Federici L Caprari C Mattei B Savino C Di Matteo A De Lorenzo G Cervone F Tsernoglou D 2001 Structural requirements of endo-polygalacturonase for the interaction with PGIP (polygalacturonase-inhibiting protein). Proceedings of the National Academy of Sciences USA 98, 13425–13430 10.1073/pnas.231473698 PMC60887 11687632

[CIT0026] Federici L Mattei B Caprari C Savino C Cervone F Tsernoglou D 1999 Crystallization and preliminary X-ray diffraction study of the endo-polygalacturonase from *Fusarium moniliforme* . Acta Crystallographica 55, 1359–1361 10.1107/s0907444999005454 10393307

[CIT0027] Ferrari S Vairo D Ausubel FM Cervone F De Lorenzo G 2003 Tandemly duplicated *Arabidopsis* genes that encode polygalacturonase-inhibiting proteins are regulated coordinately by different signal transduction pathways in response to fungal infection. Plant Cell 15, 93–106 12509524 10.1105/tpc.005165 PMC143454

[CIT0028] Genoud T Buchala AJ Chua NH Traux MJP 2002 Phytochrome signalling modulates the SA-perceptive pathway in *Arabidopsis* . The Plant Journal 31, 87–95 12100485 10.1046/j.1365-313x.2002.01338.x

[CIT0029] Gilmartin PM Sarokin L Memelink J Chua N 1990 Molecular light switches for plant genes. Plant Cell 2, 369–378 2152164 10.1105/tpc.2.5.369 PMC159894

[CIT0030] Gomathi V Gnanamanickam SS 2004 Polygalacturonase-inhibiting proteins in plant defence. Current Science 87, 1211–1217

[CIT0031] Hegedus DD Li R Buchwaldt L Parkin I Whitwill S Coutu C Bekkaoui D Rimmer SR 2008 *Brassica napus* possesses an expanded set of polygalacturonase inhibitor protein genes that are differentially regulated in response to *Sclerotinia sclerotiorum* infection, wounding and defense hormone treatment. Planta 228, 241–253 18431596 10.1007/s00425-008-0733-1

[CIT0032] James JT Dubery IA 2006 Inhibition of polygalacturonase from *Verticillium dahliae* by a polygalacturonase-inhibiting protein from cotton. Phytochemistry 57, 149–156 11382229 10.1016/s0031-9422(01)00024-3

[CIT0033] Jang S Lee B Kim C Kim S-J Yim J Han J-J Lee S Kim S-R An G 2003 The *OsFOR1* gene encodes a polygalacturonase-inhibiting protein (PGIP) that regulates floral organ number in rice. Plant Molecular Biology 53, 357–369 14750524 10.1023/b:plan.0000006940.89955.f1

[CIT0034] Janni M Bozzini T Moscetti I Volpi C D’Ovidio R 2013 Functional characterisation of wheat *Pgip* genes reveals their involvement in the local response to wounding. Plant Biology 15, 1019–1024 23574379 10.1111/plb.12002

[CIT0035] Janni M Di Giovanni M Roberti S Capodicasa C D’Ovidio R 2006 Characterization of expressed *Pgip* genes in rice and wheat reveals similar extent of sequence variation to dicot PGIPs and identifies an active PGIP lacking an entire LRR repeat. Theoretical and Applied Genetics 113, 1233–1245 16906405 10.1007/s00122-006-0378-z

[CIT0036] Kemp G Bergmann CW Clay R Van der Westhuizen AJ Pretorius ZA 2003 Isolation of a polygalacturonase-inhibiting protein (PGIP) from wheat. Molecular Plant–Microbe Interactions 16, 955–961 14601663 10.1094/MPMI.2003.16.11.955

[CIT0037] Kemp G Stanton L Bergmann CW Clay RP Albersheim P Darvill A 2004 Polygalacturonase-inhibiting proteins can function as activators of polygalacturonase. Molecular Plant–Microbe Interactions 17, 888–894 15305610 10.1094/MPMI.2004.17.8.888

[CIT0038] Kim DE Chivian D Baker D 2004 Protein structure prediction and analysis using the Robetta server. Nucleic Acids Research 32, W526–W531 15215442 10.1093/nar/gkh468 PMC441606

[CIT0039] King D Bergmann C Orlando R Benen JAE Kester HCM Visser J 2002 Use of amide exchange mass spectrometry to study conformational changes within the endopolygalacturonase II-homogalacturonan-polygalacturonase inhibiting protein system. Biochemistry 41, 10225–10233 12162737 10.1021/bi020119f

[CIT0040] Kobe B Deisenhofer J 1994 The leucine-rich repeat: a versatile binding motif. Trends in Biochemical Sciences 19, 415–421 7817399 10.1016/0968-0004(94)90090-6

[CIT0041] Kulheim C Agren J Jansson S 2002 Rapid regulation of light harvesting and plant fitness in the field. Science 297, 91–93 12098696 10.1126/science.1072359

[CIT0042] Kumar GM Mamdala P Podile AR 2009 Regulation of polygalacturonase-inhibitory proteins in plants is highly dependent on stress and light responsive elements. Plant Omics Journal 2, 238–249

[CIT0043] Leckie F Mattei B Capodicasa C Hemmings A Nuss L Aracri B De Lorenzo G Cervone F 1999 The specificity of polygalacturonase-inhibiting protein (PGIP): a single amino acid substitution in the solvent-exposed beta-strand/beta-turn region of the leucine-rich repeats (LRRs) confers a new recognition capability. EMBO Journal 18, 2352–2363 10228150 10.1093/emboj/18.9.2352 PMC1171318

[CIT0044] Lescot M Déhais P Thijs G Marchal K Moreau Y Van de Peer Y Rouzé P Rombauts S 2002 PlantCARE, a database of plant cis-acting regulatory elements and a portal to tools for *in silico* analysis of promoter sequences. Nucleic Acids Research 30, 325–327 11752327 10.1093/nar/30.1.325 PMC99092

[CIT0045] Lim J-M Aoki K Angel P Garrison D King D Tiemeyer M Bergmann C Wells L 2009 Mapping glycans onto specific *N*-linked glycosylation sites of *Pyrus communis* PGIP redefines the interface for EPG–PGIP interactions. Journal of Proteome Research 8, 673–680 19072240 10.1021/pr800855f PMC4141487

[CIT0046] Lindahl E Hess B 2001 GROMACS 3.0: a package for molecular simulation and trajectory analysis. Journal of Molecular Modeling 7, 306–317

[CIT0047] Lu L Zhou F Zhou Y Fan X Ye S Wang L Chen H Lin Y 2012 Expression profile analysis of the polygalacturonase-inhibiting protein genes in rice and their responses to phytohormones and fungal infection. Plant Cell Reports 31, 1173–1187 22362377 10.1007/s00299-012-1239-7

[CIT0048] Lyskov S Gray JJ 2008 The RosettaDock server for local protein–protein docking. Nucleic Acids Research 36, W233–W238 18442991 10.1093/nar/gkn216 PMC2447798

[CIT0049] Marchler-Bauer A Lu S Anderson JB, 2011 CDD: a Conserved Domain Database for the functional annotation of proteins. Nucleic Acids Research 39, D235–D229 21109532 10.1093/nar/gkq1189 PMC3013737

[CIT0050] Matsushima N Miyashita H 2012 Leucine-rich repeat (LRR) domains containing intervening motifs in plants. Biomolecules 2, 288–311 24970139 10.3390/biom2020288 PMC4030839

[CIT0051] Mattei B Bernalda MS Federici L Roepstorff P Cervone F Boffi A 2001 Secondary structure and post-translational modifications of the leucine-rich repeat protein PGIP (polygalacturonase-inhibiting protein) from *Phaseolus vulgaris* . Biochemistry 40, 569–576 11148052 10.1021/bi0017632

[CIT0052] Maulik A Ghosh H Basu S 2009 Comparative study of protein-protein interaction observed in Polygalacturonase-inhibiting proteins from *Phaseolus vulgaris* and *Glycine max* and Polygalacturonase from *Fusarium moniliforme* . BMC Genomics 10, S19 19958482 10.1186/1471-2164-10-S3-S19 PMC2788371

[CIT0053] Misas-Villamil JC Van der Hoorn RAL 2008 Enzyme–inhibitor interactions at the plant–pathogen interface. Current Opinion in Plant Biology 11, 380–388 18550418 10.1016/j.pbi.2008.04.007

[CIT0054] Mohnen D 2008 Pectin structure and biosynthesis. Current Opinion in Plant Biology 11, 266–277 18486536 10.1016/j.pbi.2008.03.006

[CIT0055] Mullineaux P Ball L Escobar C Karpinska B Creissen G Karpinski S 2000 Are diverse signaling pathways integrated in the regulation of *Arabidopsis* antioxidant defence gene expression in response to excess excitation energy? Philosophical Transactions of the Royal Society B 355, 1531–1540 10.1098/rstb.2000.0713 PMC1692875 11128006

[CIT0056] Nakai K Horton P 1999 PSORT: a program for detecting sorting signals in proteins and predicting their subcellular localization. Trends in Biochemical Sciences 24, 34–35 10087920 10.1016/s0968-0004(98)01336-x

[CIT0057] Notredame C Higgins DG Heringa J 2000 T-coffee: a novel method for fast and accurate multiple sequence alignment. Journal of Molecular Biology 302, 205–217 10964570 10.1006/jmbi.2000.4042

[CIT0058] Petersen TN Brunak S Von Heijne G Nielsen H 2011 SignalP 4.0: discriminating signal peptides from transmembrane regions. Nature Methods 8, 785–786 21959131 10.1038/nmeth.1701

[CIT0059] Pettersen EF Goddard TD Huang CC Couch GS Greenblatt DM Meng EC Ferrin TE 2004 UCSF Chimera—a visualization system for exploratory research and analysis. Journal of Computational Chemistry 25, 1605–1612 15264254 10.1002/jcc.20084

[CIT0060] Pickersgill R Smith D Worboys K Jenkins J 1998 Crystal structure of polygalacturonase from *Erwinia carotovora* ssp. *carotovora* . Journal of Biological Chemistry 273, 24660–24664 9733763 10.1074/jbc.273.38.24660

[CIT0061] Raiola A Sella L Castiglioni C Balmas V Favaron F 2008 A single amino acid substitution in highly similar endo-PGs from *Fusarium verticillioides* and related *Fusarium* species affects PGIP inhibition. Fungal Genetics and Biology 45, 776–789 18171630 10.1016/j.fgb.2007.11.003

[CIT0062] Rodrigues PGLM Levitt M Chopra G 2012 KoBa^MIN^: a knowledge-based minimization web server for protein structure refinement. Nucleic Acids Research 40, 323–328 22564897 10.1093/nar/gks376 PMC3394243

[CIT0063] Sambrook J Russell DW 2001 Molecular cloning: a laboratory manual. Argentine J Irwin N Janssen KA Curtis S Zierler M, eds. New York: Cold Spring Harbor Laboratory Press

[CIT0064] Schacht T Unger C Pich A Wydra K 2011 Endo- and exo-polygalacturonases of *Ralstonia solanacearum* are inhibited by polygalacturonase-inhibiting protein (PGIP) activity in tomato stem extracts. Plant Physiology and Biochemistry 49, 377–387 21367611 10.1016/j.plaphy.2011.02.001

[CIT0065] Sehgal D Rajaram V Armstead IP Vadez V Yadav YP Hash CT Yadav RS 2012 Integration of gene-based markers in a pearl millet genetic map for identification of candidate genes underlying drought tolerance quantitative trait loci. BMC Plant Biology 12, 9 22251627 10.1186/1471-2229-12-9 PMC3287966

[CIT0066] Sella L Castiglioni C Roberti S D’Ovidio R Favaron F 2004 An endo-polygalacturonase (PG) of *Fusarium moniliforme* escaping inhibition by plant polygalacturonase-inhibiting proteins (PGIPs) provides new insights into the PG–PGIP interaction. FEMS Microbiology Letters 240, 117–124 15500988 10.1016/j.femsle.2004.09.019

[CIT0067] Shanmugam V 2005 Role of extracytoplasmic leucine rich repeat proteins in plant defence mechanisms. Microbiological Research 160, 83–94 15782942 10.1016/j.micres.2004.09.014

[CIT0068] Shivashankar S Thimmareddy C Roy TK 2010 Polygalacturonase inhibitor protein from fruits of anthracnose resistant and susceptible varieties of chilli (*Capsicum annuum* L). Indian Journal of Biochemistry and Biophysics 47, 243–248 21174952

[CIT0069] Sicilia F Fernandez-Recio J Caprari C De Lorenzo G Tsernoglou D Cervone F Federici L 2005 The polygalacturonase-inhibiting protein PGIP2 of *Phaseolus vulgaris* has evolved a mixed mode of inhibition of endopolygalacturonase PG1 of *Botrytis cinerea* . Plant Physiology 139, 1380–1388 16244152 10.1104/pp.105.067546 PMC1283773

[CIT0070] Singh SD 1995 Downy mildew of pearl millet. Plant Disease. 79, 545–549 10.1094/PDIS.1998.82.7.791 30856952

[CIT0071] Spinelli F Mariotti L Mattei B Salvi G Cervone F Caprari C 2009 Three aspartic acid residues of polygalacturonase-inhibiting protein (PGIP) from *Phaseolus vulgaris* are critical for inhibition of *Fusarium phyllophilum* PG. Plant Biology 11, 738–743 19689781 10.1111/j.1438-8677.2008.00175.x

[CIT0072] Stotz HU Bishop JG Bergmann CW Koch M Albersheim P Darvill AG Labavitch JM 2000 Identification of target amino acids that affect interactions of fungal polygalacturonases and their plant inhibitors. Physiological and Molecular Plant Pathology 56, 117–130

[CIT0073] Studier FW 2005 Protein production by auto-induction in high-density shaking cultures. Protein Expression and Purification 41, 207–234 15915565 10.1016/j.pep.2005.01.016

[CIT0074] Talavera G Castresana J 2007 Improvement of phylogenies after removing divergent and ambiguously aligned blocks from protein sequence alignments. Systematic Biology 56, 564–577 17654362 10.1080/10635150701472164

[CIT0075] Tina KG Bhadra R Srinivasan N 2007 PIC: Protein Interactions Calculator. Nucleic Acids Research 35, W473–W476 17584791 10.1093/nar/gkm423 PMC1933215

[CIT0076] Tovchigrechko A Vakser IA 2006 GRAMM-X public web server for protein–protein docking. Nucleic Acids Research 34, W310–W314 16845016 10.1093/nar/gkl206 PMC1538913

[CIT0077] Unni S Huang Y Hanson RM Tobias M Krishnan S Li WW Nielsen JE Baker NA 2011 Web servers and services for electrostatics calculations with APBS and PDB2PQR. Journal of Computational Chemistry 32, 1488–1491 21425296 10.1002/jcc.21720 PMC3062090

[CIT0078] Van den Brink J De Vries RP 2011 Fungal enzyme sets for plant polysaccharide degradation. Applied Microbiology and Biotechnology 91, 1477–1492 21785931 10.1007/s00253-011-3473-2 PMC3160556

[CIT0079] Vorwerk S Somerville S Somerville C 2004 The role of plant cell wall polysaccharide composition in disease resistance. Trends in Plant Science 9, 203–209 15063871 10.1016/j.tplants.2004.02.005

[CIT0080] Wagenknecht M Meinhardt F 2011 Copy number determination, expression analysis of genes potentially involved in replication, and stability assays of pAL1—the linear megaplasmid of *Arthrobacter nitroguajacolicus* Ru61a. Microbiological Research 166, 14–26 20116226 10.1016/j.micres.2009.12.005

[CIT0081] Wang W Barnaby JY Tada Y Li H Tor M Caldelari D Lee D Fu X-D Dong X 2011 Timing of plant immune responses by a central circadian regulator. Nature 470, 110–114 21293378 10.1038/nature09766 PMC6601609

