# Supplementary material for: Experimental and bioinformatic characterization of a recombinant polygalacturonase-inhibitor protein from pearl millet and its interaction with fungal polygalacturonases
Source: J Exp Bot. 2014 Jun 30;65(17):5033–47. doi: 10.1093/jxb/eru266 (PMC4144779; doi:10.1093/jxb/eru266)
Supplement: Supplementary Data [file supp_65_17_5033__index.html]

Experimental and bioinformatic characterization of a recombinant polygalacturonase-inhibitor protein from pearl millet and its interaction with fungal polygalacturonases — Experimental and bioinformatic characterization of a recombinant polygalacturonase-inhibitor protein from pearl millet and its interaction with fungal polygalacturonases — Experimental and bioinformatic characterization of a recombinant polygalacturonase-inhibitor protein from pearl millet and its interaction with fungal polygalacturonases — Supplementary Data 

# Experimental and bioinformatic characterization of a recombinant polygalacturonase-inhibitor protein from pearl millet and its interaction with fungal polygalacturonases

## Supplementary Data

Data files

**Files in this Data Supplement:**

- Supplementary Data - Supplementary Data
