# Supplementary material for: Experimental and bioinformatic characterization of a recombinant polygalacturonase-inhibitor protein from pearl millet and its interaction with fungal polygalacturonases
Source: J Exp Bot. 2014 Jun 30;65(17):5033–47. doi: 10.1093/jxb/eru266 (PMC4144779; doi:10.1093/jxb/eru266)
Supplement: Supplementary Data [file supp_eru266_jexbot117093_file001.pdf]

Journal of Experimental Botany

Experimental and bioinformatic characterisation of a recombinant polygalacturonase-inhibitor protein from pearl millet and its interaction with fungal polygalacturonases

S. Ashok Prabhu, Ratna Singh, Stephan Kolkenbrock, Sujeeth Neerakkal, Nour Eddine El Gueddari, Bruno M. Moerschbacher, Ramachandra K. Kini, Martin Wagenknecht

Supplementary Table S1. List of plant PGIPs used in the protein phylogenetic analysis. The protein sequences were retrieved from the GenBank database, NCBI.

	Species name	Protein	Protein accession number
	Brachypodium distachyon	PGIP	XP_003569139
	Brachypodium sylvaticum	PGIP	ABL85038
	Cenchrus americanus	PGIP1	AFA28255
	Hordeum vulgare	PGIP	BAK00081
	Oryza sativa	PGIP1	AM180652
	Oryza sativa	PGIP2	AM180653
	Oryza sativa	PGIP3	AM180654
	Oryza sativa	PGIP4	AM180655
	Oryza sativa	FOR1	AF466357
	Setaria italica	PGIP	XP_004960589
	Sorghum bicolor	PGIP	XP_002439099
	Triticum aestivum	PGIP1	AM180656
	Triticum aestivum	PGIP2	AM180657
	Triticum durum	PGIP1	CAP07571
	Triticum monococcum	PGIP	CAJ55697
	Zea mays	PGIP	NP_001147231
Dicot	*Arabidopsis thaliana*	PGIP1	AF229249
	Arabidopsis thaliana	PGIP2	AF229250
	Brassica napus	PGIP	AAM95647
	Carica papaya	PGIP	ADV16115
	Chorispora bungeana	PGIP	ACA66126
	Citrus iyo	PGIP	BAA31843
	Cucumis melo	PGIP	AAP41199
	Eucalyptus grandis	PGIP	AF159167
	Glycine max	PGIP1	AJ972660
	Glycine max	PGIP2	AJ972661
	Glycine max	PGIP3	AJ972662
	Glycine max	PGIP4	AJ972663
	Gossypium barbadense	PGIP	AAQ19807
	Gossypium hirsutum	PGIP	ACD93187
	Malus hupehensis	PGIP	ACJ65215
	Phaseolus vulgaris	PGIP1	AJ786408
	Phaseolus vulgaris	PGIP2	AJ786409
	Phaseolus vulgaris	PGIP3	AJ786410
	Phaseolus vulgaris	PGIP4	AJ786411
	Prunus salicina	PGIP	ACY41032
	Pyrus pyrifolia	PGIP	ACY56891
	Raphanus sativus	PGIP	ABN50914
	Solanum lycopersicum	PGIP	XP_004244078
	Solanum torvum	PGIP	ACR19029
	Vitis thunbergii	PGIP	ABU82741
	Vitis vinifera	PGIP	AF499451

Supplementary Table S2. Construction of the *Pgl*PGIP1, vector control and *Fm*PGIII expression plasmids for expression in *Escherichia coli* SHuffle® T7 Express [pLysSRARE2]. The steps of the cloning strategy used to obtain the expression cassettes are summarised in the table.

**Expression construct code**	**Primer sequences (5'-3') (Forward/Reverse)**	**Cloning strategy/Construct representation**
(A)	GGAATTCCATATGTCGCCCATACG CTGC/ CGGGATCCCGCTTGCAGAGATTAG CAAGC	The mature pearl millet PGIP sequence was PCR-amplified using pearl millet genomic DNA as template, amplicon cut with *Nde*I, *Bam*HI and ligated with pET-22b(+), cut with the same enzymes. **pET-22b-*Pgl*PGIP1-6xHis** (6xHis: hexa histidine tag)
(B)	GCTCTAGAAATAATTTTGTTTAAG AAGGAGATATAATTATGAAAATCGA AGAAGGTAAAC/ GGAATTCCATATGCCTTCCCTCGA TCCCG	The maltose-binding protein (MBP) coding sequence along with the one for the Factor Xa protease cleavage site (IEGR) were PCR-amplified using pMAL-c2x as template. The amplicon was digested with *Nde*I, *Xba*I and sub-cloned in the vector backbone of construct (A) digested with the same enzymes. **pET-22b-MBP-IEGR-*Pgl*PGIP1-6xHis**
(C)	GCTCTAGAAATAATTTTGTT TAAG AAGGAGATATAATTATGTGGTCACA TCCTCAATTTGAAAAAATGAAAATC GAAGAAGGTAAACTGG/ GGAATTCCATATGCCTTCCCTCGA TCCCG	A Strep-tag® II was introduced N-terminal of MBP by PCR using (B) as template. The amplicon was cut with *Nde*I, *Xba*I and sub-cloned in the vector backbone of construct (B) digested with the same enzymes. **pET-22b-Strep-tag® II-MBP-IEGR-*Pgl*PGIP1-6xHis**
(D)	AGGATGTGACCAGTGGTGGT GGT GGTGGTGCTC/ CAATTTGAAAAATAGGATCCGGC TGCTAACAA AG	An additional Strep-tag® II was introduced C-terminal of MBP by PCR using (C) as template. The amplicon was recircularised by ligation. **pET-22b-Strep-tag® II-MBP-IEGR-*Pgl*PGIP1-6xHis-Strep-tag® II**
(E)	ATGAAAATCGAAGAAGGTAAACT GG/ AATTATATCTCCTTCTTAAACAAA ATTATTTCT AGAGG	The N-terminal Strep-tag® II of construct (D) was eliminated by PCR using (D) as template to retain the Strep-tag® II only C-terminal of PGIP. The amplicon was recircularised by ligation. **pET-22b-MBP-IEGR-*Pgl*PGIP1-6xHis-Strep-tag® II**
(F)	ATGCCTTCCCTCGATCCCG/ CGGGATCCGAATTCGAGCTC	The vector control was generated by eliminating the PGIP sequence from construct (E) by PCR using (E) as template. The amplicon was recircularised by ligation. **pET-22b-MBP-IEGR-6xHis-Strep-tag® II**
(G)	GGAATTCCCATATGGATGCTTGCA CCGTGAC TG/ CGAGCTCGCTGGGGCAAGTGTTGC	pGEMT containing *Fm*PGIII (kind gift from Prof. Francesco Favaron, Dip. Territorio e Sistemi agro-forestali, sez. Patologia Vegetale, Universita degli Studi di Padova, Viale dell_Universita 16, I-35020 Legnaro, Italy) was PCR-amplified, digested with *Nde*I and *Sac*I to release the *Fm*PGIII fragment, which was sub-cloned in pET-22b(+)-Strep-tag® II (C-terminal) digested with the same enzymes. **pET-22b-*Fm*PGIII-Strep-tag® II**

Supplementary Table S3. Upstream sequence analysis of the *Pglpgip1* gene. Important *cis*-regulatory elements present in the nucleotide sequence upstream of *Pglpgip1* open reading frame with their consensus sequence and functional description retrieved from an analysis using PlantCARE.

cis element	Sequence	Function assigned
	**Core promoter elements**	
TATA-box	TATATATA; TAATA	Core promoter element
CAAT-box	CAAAT; gGCAAT; CCAAT; CAATT; CAAT	Common *cis*-acting element in promoter and enhancer regions
	**Light responsive elements**	
GA-motif	ATAGATAA	Part of a light responsive element
GAG-motif	AGAGATG; GGAGATG	Part of a light responsive element
GATA-motif	AAGATAAGATT	Part of a light responsive element
TCT-motif	TCTTAC	Part of a light responsive element
Box4	ATTAAT	Part of a light responsive element
Box1	TTTCAAA	Light responsiveness
Sp1 (Specificity protein 1)	CC(G/A)CCC	Light responsiveness
G-Box	CACGTA; TACGTG; CACGTC	Light responsiveness
	**Defence/stress responsive elements**	
ABRE (ACGT-containing abscisic acid response element)	TACGTG	Abscisic acid responsiveness
TC-rich repeats	ATTTTCTCCA	Defence and stress responsiveness
TCA-element	GAGAAGAATA	Salicylic acid responsiveness
TGACG-motif	TGACG	Methyl jasmonate-responsiveness
CGTCA-motif	CGTCA	Methyl jasmonate-responsiveness
ERE (Ethylene response element)	ATTTCAAA	Ethylene responsiveness
	**Other responsive elements**	
ARE (Anaerobic response element)	TGGTTT	Essential for the anaerobic induction
GC-motif	CCCCCG	Enhancer-like element involved in anoxic specific inducibility
GCN4_motif	TGAGTCA	Endosperm expression
Skn-1_motif	GTCAT	Endosperm expression

Supplementary Table S4. Refinement of *Pgl*PGIP1 and *Fm*PGIII models. Structural models were refined using the KoBaMIN server and further geometric accuracy of the constructed models was evaluated using MolProbity program.

**Parameters**	***Pgl*PGIP1**	***Fm*PGIII**
$KoBa^{MIN}$ potential energy	-7076.7375	-6975.7462
Clash score all atoms	6.67	7.02
Poor rotamers	1.59%	1.04%
Ramachandran outliers	0.32%	0.86%
Ramachandran favoured	96.12%	94.54%
CB deviation >0.25Å	0	0
Residues with bad bonds	0.00%	0.00%
Residues with bad angles	0.00%	0.00%
MolProbity score	1.78 (86%)	1.77 (86%)

Supplementary Table S5. Docking and interface scores of *Pgl*PGIP1:*An*PGII and *Pgl*PGIP1:*Fm*PGIII complexes.

Docked complexes	Docking score	Interface score
*Pgl*PGIP1:*An*PGII	-610	-10.18
*Pgl*PGIP1:*Fm*PGIII	-579	-5.88

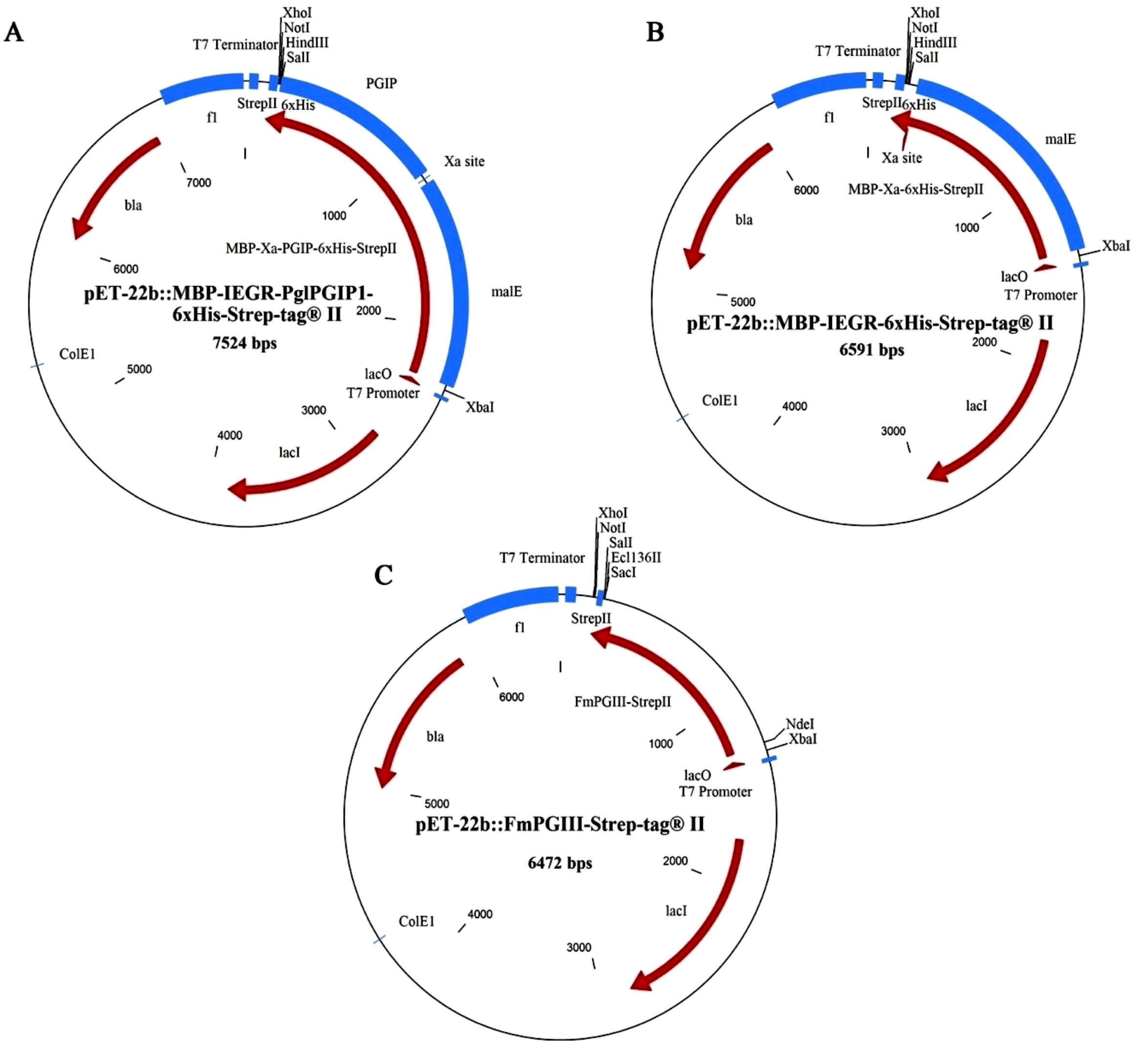

Supplementary Fig. S1. Pictorial representation of the *Pgl*PGIP1 (A), vector control (B) and *Fm*PGIII (C) expression plasmids for expression in *Escherichia coli* SHuffle® T7 Express [pLysSRARE2]. The details of the steps involved in their construction are provided in **Supplementary Table S2.**

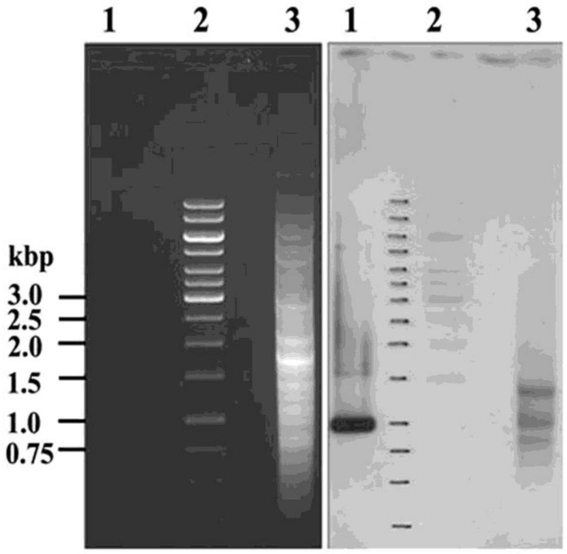

Supplementary Fig. S2. Southern blot analysis of pearl millet total DNA. (A) Total pearl millet DNA was digested with restriction endonuclease *Apo*I, separated on 0.7% agarose gel (left panel), and transferred onto a positively charged nylon membrane. The DNA was hybridised with a probe deduced and PCR-amplified from the cloned *PglPGIP1p* gene. The right panel displays the corresponding exposed X-ray film. To ensure conspicuousness of the DNA size standard, positions of the marker bands were indicated on the membrane after the transfer; then membrane and exposed film were merged and the bands were marked on the film as well. Lane 1. 250 pg of unlabelled probe fragment (hybridisation control); 2. DNA size standard (GeneRuler™ 1kb DNA Ladder, Fermentas); 3. 5 μg of *Apo*I-digested total pearl millet DNA.

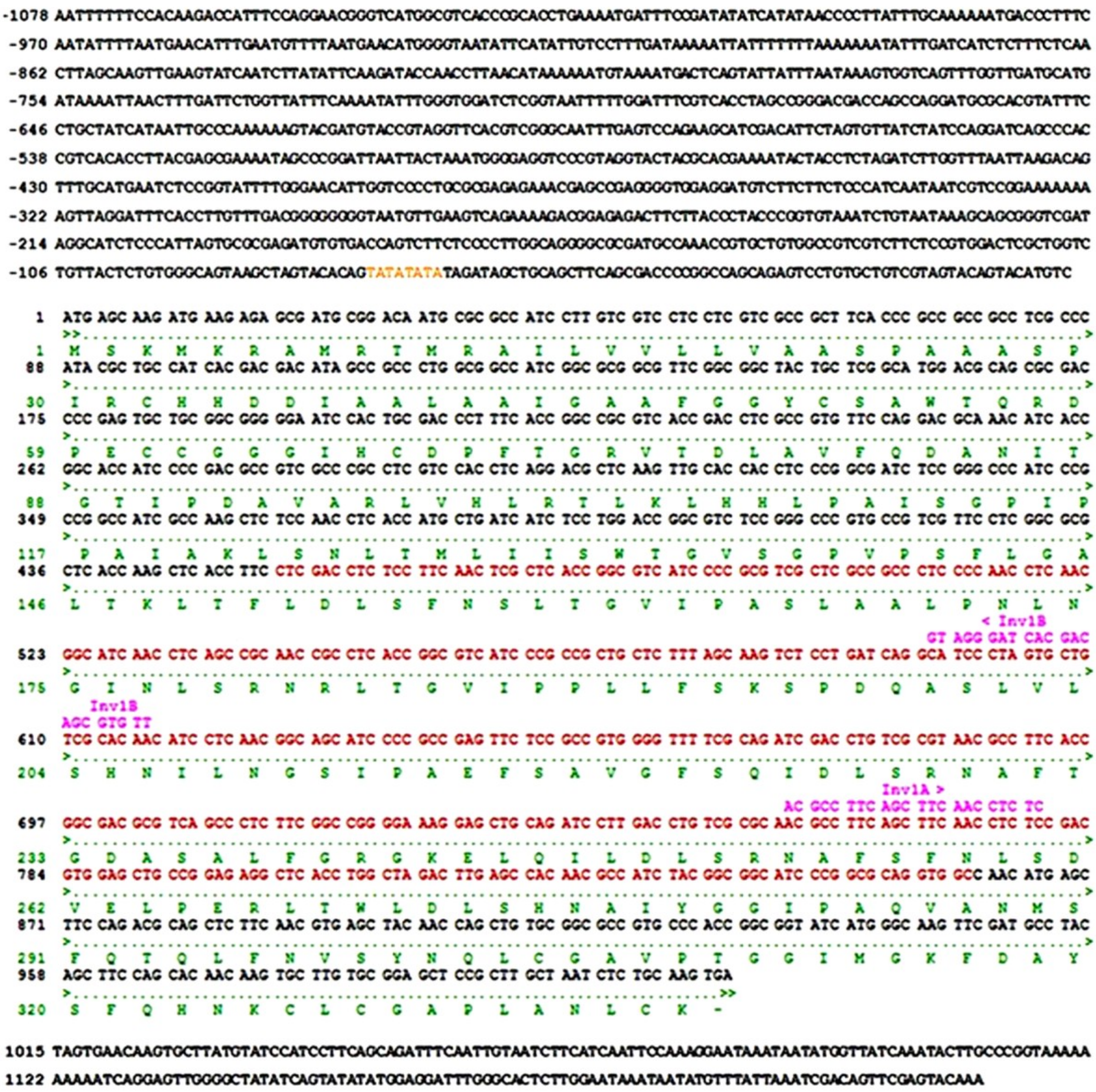

Supplementary Fig. S3. Nucleotide and derived amino acid sequences of the pearl millet *Pglpgip1* gene. Nucleotides are numbered from the start codon (+1) shown in black and amino acid numbering is shown in green. A putative TATA box is shown (in orange) in the sequence upstream of the start codon. The partial pearl millet *Pglpgip1p* nucleotide sequence based on which the inverse PCR was carried out is shown in dark brown. The sequence and orientation of the primers used in the inverse PCR are shown in purple.

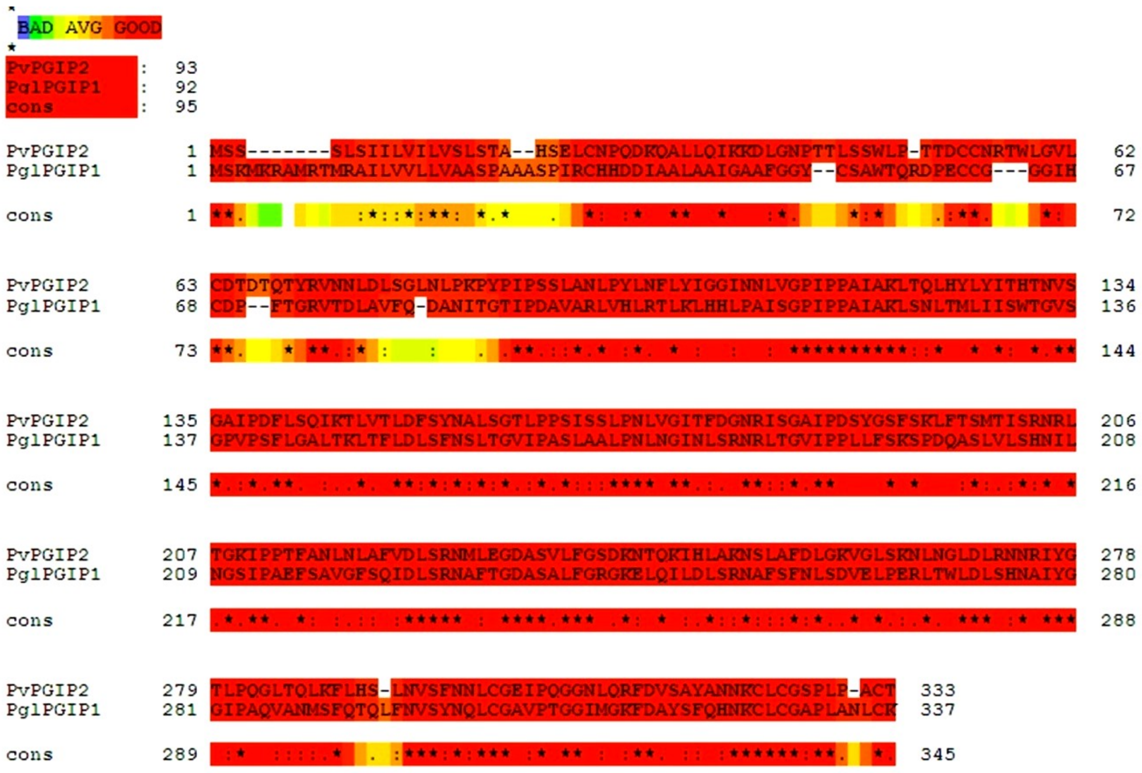

Supplementary Fig. S4. Alignment of *Pgl*PGIP1 and *Pv*PGIP2 sequences using T-Coffee multi-alignment tool. The alignment colour scale and scores are specified.

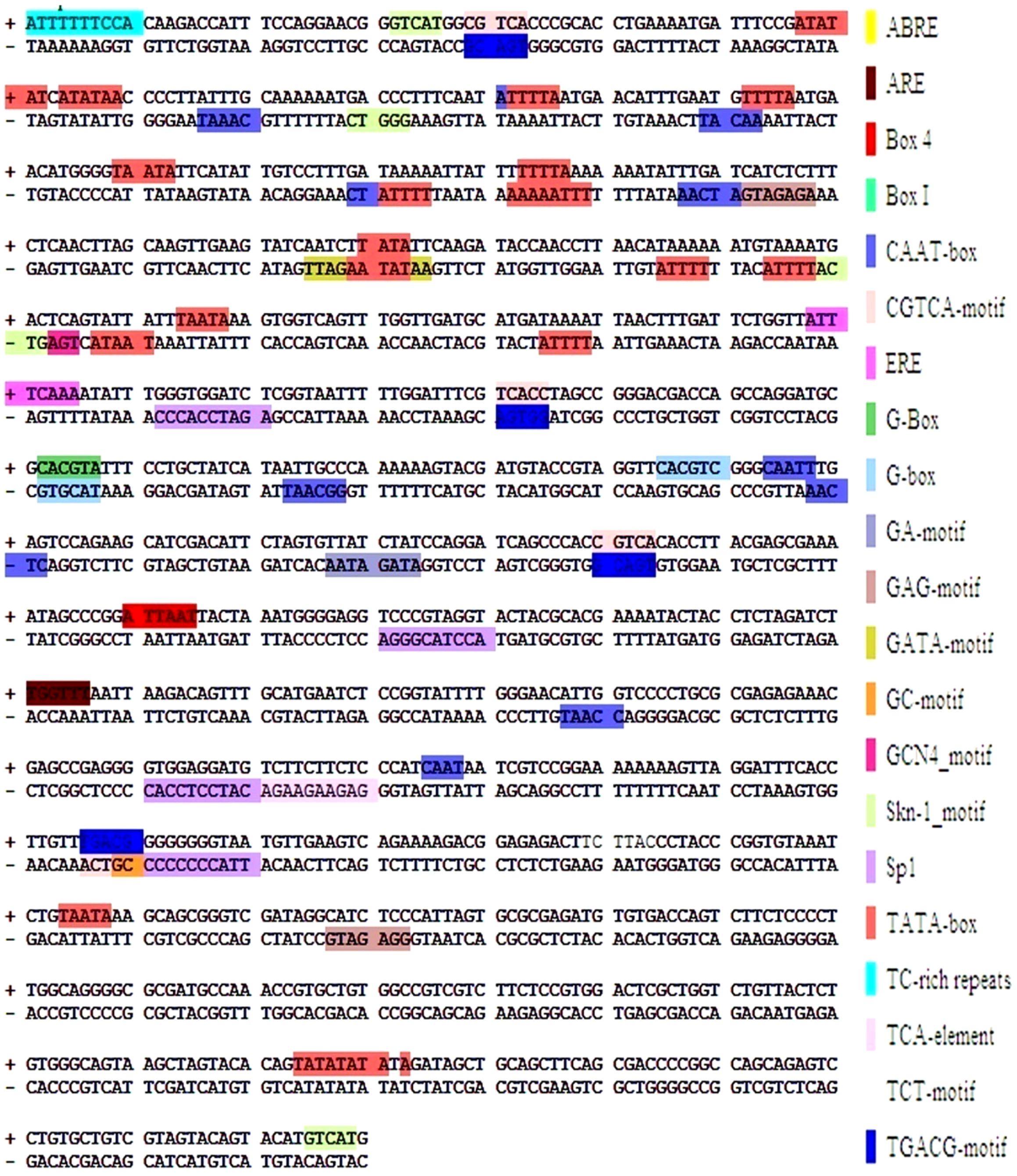

Supplementary Fig. S5. Upstream *cis*-regulatory elements in the *Pglpgip1* gene. Nucleotide sequences of important *cis*-regulatory elements present upstream of the *Pglpgip1* open reading frame with their consensus sequence retrieved from PlantCARE, an online tool used for the analysis. The colouring scheme used to represent the different *cis*-reulatory elements are given on the right side. The '+' and '-' refer to nucleotide strand orientations. Sp1, Specificity protein1; ABRE, ACGT-containing abscisic acid response element; ERE, Ethylene response element; ARE, Anaerobic response element.

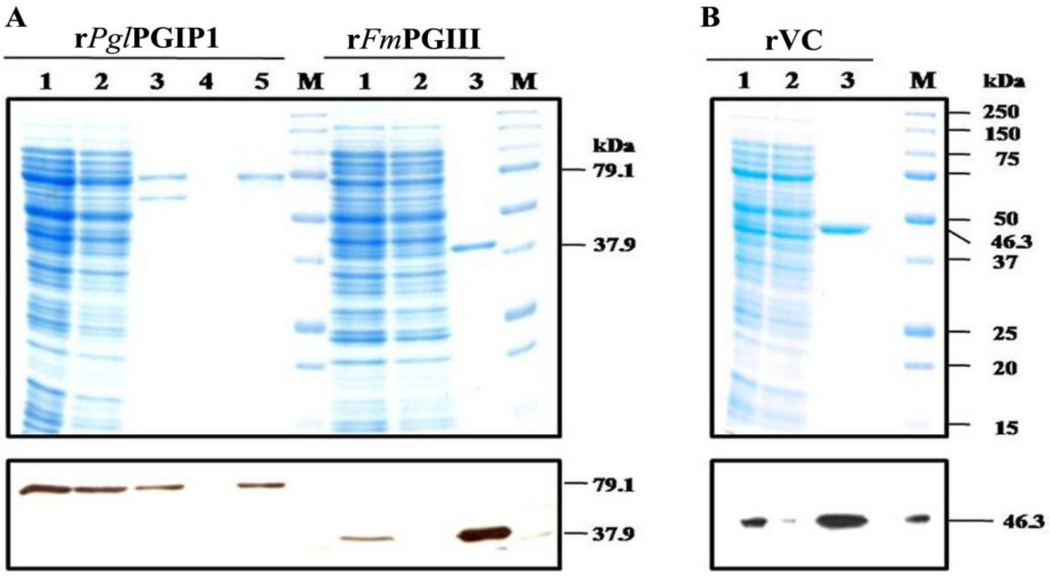

Supplementary Fig. S6. Purification of recombinant *Pgl*PGIP1, vector control and *Fm*PGIII fusion proteins synthesised in *Escherichia coli* SHuffle® T7 Express [pLysSRARE2]. SDS-PAGE profile of various protein purification fractions and their corresponding Western blots to track the recombinant proteins using Strep-Tactin®-HRP-conjugates (IBA-lifesciences) for chemiluminescence detection. (A) r*Pgl*PGIP1purification. Lane 1. Total protein extract from *E. coli* expressing recombinant *Pglpgip1*, 2. Strep-Tactin column flow-through, 3. Strep-Tactin column eluate, 4. RESOURCE Q column flow-through, 5. RESOURCE Q column eluate (purified r*Pgl*PGIP1). r*Fm*PGIII purification. Lane 1. Total protein extract from *E. coli* expressing recombinant *FmPGIII*, 2. Strep-Tactin column flow-through, 3. Strep-Tactin column eluate (purified r*Fm*PGIII). (B) rVC purification. Lane 1. Total protein extract from *E. coli* expressing the vector control, 2. Strep-Tactin column flow-through, 3. Strep-Tactin column eluate (purified rVC). Lane M. Precision Plus All Blue Protein Molecular Weight Standard (Bio-Rad).

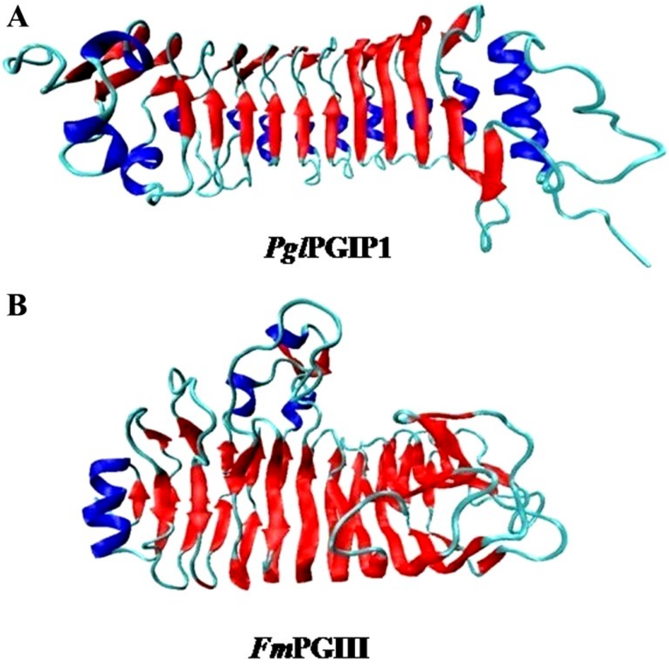

Supplementary Fig. S7. Homology modelling of *Pgl*PGIP1 and *Fm*PGIII. The homology models (ribbon representations) of *Pgl*PGIP1 (A) and *Fm*PGIII (B) were generated using already known structures of *Pv*PGIP2 (1OGQ.pdb) and *Fm*PGI (1HG8.pdb) serving as templates, respectively. The initial models were generated using MODELLER 9.12 package. The quality of the generated models was assessed using Verify3D server. Post-refinement of structural models was carried out using the KoBa^{MIN} server and further geometric accuracy of the constructed models was evaluated using MolProbity 4.02b program.
